# The hook-like adaptor and cargo-binding (HAC) domain in the kinesin-2 tail enables adaptor assembly and cargo recognition

**DOI:** 10.1126/sciadv.ady5861

**Published:** 2025-10-24

**Authors:** Xuguang Jiang, Radostin Danev, Sotaro Ichinose, Baichun Niu, Sumio Ohtsuki, Haruaki Yanagisawa, Satoru Nagatoishi, Kouhei Tsumoto, Nobutaka Hirokawa, Masahide Kikkawa

**Affiliations:** ^1^ Department of Cell Biology and Anatomy, Graduate School of Medicine, The University of Tokyo, Tokyo 113-0033, Japan.; ^2^ Department of Anatomy, Graduate School of Medicine, Gunma University, Gunma 371-8511, Japan.; ^3^ Department of Pharmaceutical Microbiology, Faculty of Life Sciences, Kumamoto University, Kumamoto 862-0973, Japan.; ^4^ Department of Bioengineering, School of Engineering, The University of Tokyo, Tokyo 113-8656, Japan.; ^5^ Graduate School of Medicine, Juntendo University, Tokyo 113-8421, Japan.

## Abstract

Intracellular transport relies on motor proteins such as kinesins to deliver cargo along microtubules, yet how they recognize cargo remains unclear. Here, we present high-resolution cryo–electron microscopy structures of the heterotrimeric kinesin-2 complex (KIF3A/KIF3B/KAP3) bound to the cargo protein APC. Our findings reveal a previously uncharacterized KIF3 tail hook-like motif, termed the “HAC” domain, which mediates binding to both KAP3 adaptor and APC cargo. Within this domain, the KIF3A helical regions ensure cargo specificity, while a β-hairpin and KIF3B provide structural support. Biochemical and neuronal experiments confirm its functional importance. Notably, the HAC/KAP3 structure resembles hook-like architectures seen in kinesin-1 and dynein, suggesting a shared cargo recognition framework. These findings also shed light on kinesin-2 cargo specificity and offer a structural framework for understanding related neuronal transport mechanisms.

## Teaser

A previously unidentified structural motif in kinesin-2 sheds light on selective cargo transport in neurons.

## INTRODUCTION

The molecular mechanisms orchestrating the dynamic regulation of motor protein-driven intracellular transport have been a focal point of scientific inquiry for several decades (*1*–*4*). A key class of motor proteins, the kinesin superfamily proteins (KIFs), plays a pivotal role in microtubule-based intracellular trafficking by recognizing and transporting various cargoes essential for diverse cellular processes (*5*) Kinesin tails interact with cargoes through adaptors or accessory proteins that mediate specificity and control; however, because of the disordered nature of the kinesin tail region, which imparts high structural flexibility, the molecular basis for kinesin-mediated cargo recognition and binding remains an unresolved question (*4*–*7*). Recent small-angle x-ray scattering and negative-stain electron microscopy (EM) studies have revealed low-resolution binding models of kinesins and adaptors (*8*, *9*), and crystal studies of kinesin-1 light chain and cargo peptides have provided valuable insights into direct kinesin-cargo interactions (*10*–*12*), but these insights remain limited. Heterotrimeric kinesin-2 (KIF3/KAP3 in mammals) is one of the most widely expressed and abundant kinesins (*13*–*15*) functioning as a complex of heterodimeric KIF3 motor (KIF3A/KIF3B or KIF3A/KIF3C) and the armadillo repeat (ARM)–containing adaptor KAP3 (*16*–*18*). Beyond its essential role in intraflagellar transport (IFT) (*15*, *19*, *20*), KIF3/KAP3 has also been implicated in transporting diverse intracellular cargos (*21*–*28*). Notably, the tumor suppressor protein adenomatous polyposis coli (APC) has been identified as a KIF3/KAP3 cargo involved in RNA and vehicle transport crucial for neurons (*27*, *29*, *30*), accounting for ~12% of KIF3-associated organelles (*31*). However, the mechanism of KIF3/KAP3 cargo recognition remains largely unclear.

To bridge this gap, we used cryo–electron microscopy (cryo-EM) to obtain high-resolution structural analysis of the KIF3A/B/KAP3-APC complex (*9*, *15*–*18*, *27*, *29*–*31*), providing the first detailed structural insight into the kinesin-adaptor-cargo system. This structural study reveals how the kinesin tail regions of KIF3A and KAP3 adaptor cooperate to mediate cargo recognition while also highlighting isoform-specific differences in kinesin-2 function. Our findings uncover a structural feature, the hook-like adaptor and cargo-binding (HAC) domain with helix-β-hairpin-helix motifs, in the kinesin-2 tail, coordinating adaptor and cargo specificity. Biochemical and cellular results validated our structural findings and confirmed the functional importance. Notably, the structural similarities between the dynein FTS-HOOK-FHIP1B (FHF) complex (*32*, *33*) and the kinesin-1 heavy chain/light chain cargo-binding region (*8*) with the KIF3 tail/KAP3 structure highlight the potential similarity of this mechanism.

## RESULTS

### H-βh-H motif in the KIF3 tail serves as a scaffold for KAP3 interaction

**Fig. 1.**
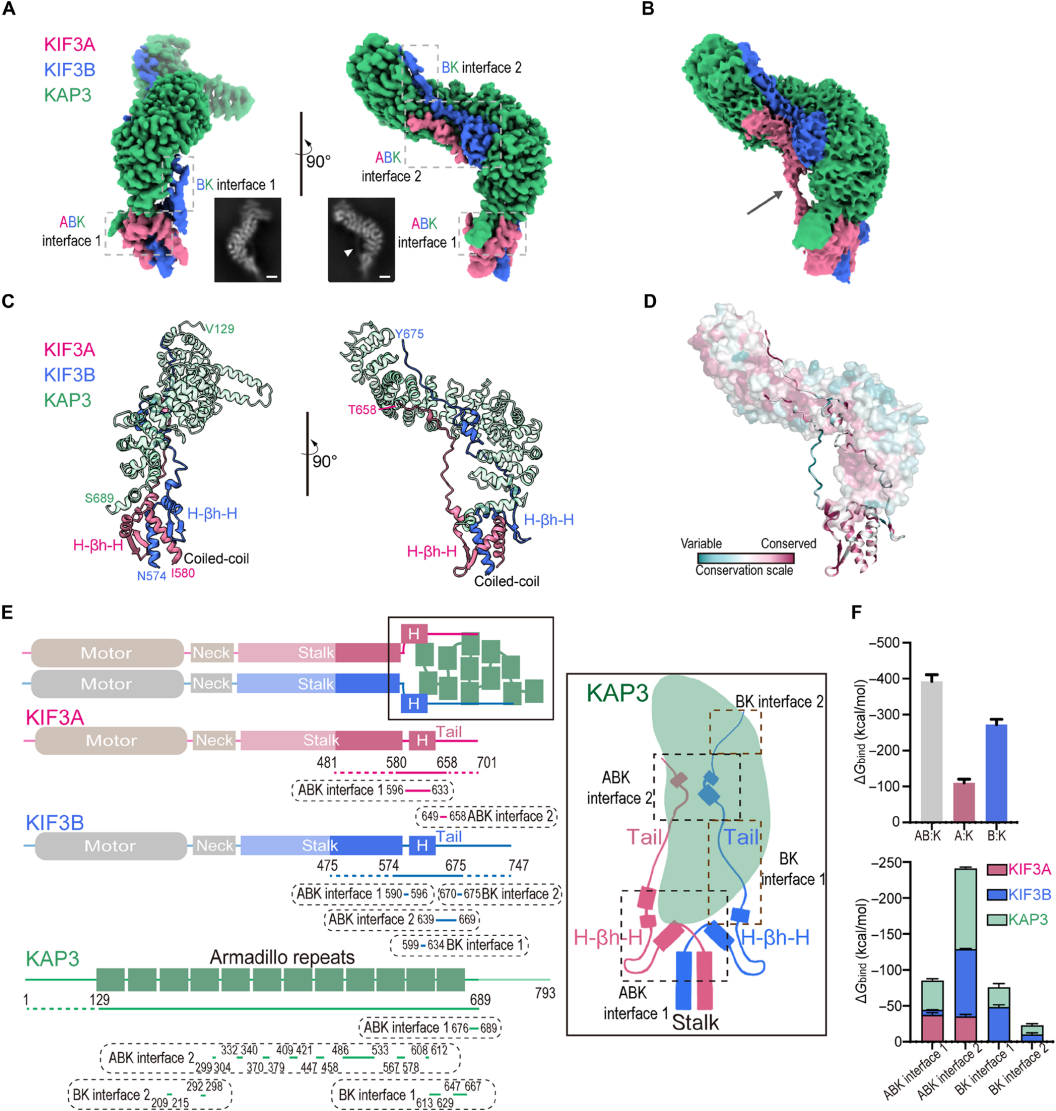
Basic architecture of the KIF3A/B tail/KAP3 complex. (**A**) Cryo-EM map of the ABK complex. The interfaces are indicated by dashed-line boxes. Two different orientation views are shown, whose relative rotation is indicated. The insets show representative two-dimensional (2D) averages (The 2D averages are not exactly the same orientation as the 3D maps). The white arrowhead in the 2D image points to the KIF3A-linking loop, which is also visible in panel B. Scale bars for 2D views, 2 nm. (**B**) Volume of the KIF3A tail–linking loop, which is visible in the raw EM map at a higher contour threshold, as indicated by an arrow. (**C**) Reconstructed structural model of the ABK complex comprising the coiled-coiled stalk, the H-βh-H motif, and the extended tail loops. (**D**) Conservation analysis of the ABK model. (**E**) Diagrams illustrating the KIF3/KAP3 binding mode. In the 2D schematic, the transparent regions represent areas not included in the KIF3/KAP3 constructs. For the constructs, KIF3A (residues 481 to 701), KIF3B (residues 475 to 747), and KAP3 (residues 1 to 693), the visible portions in the structure are shown with solid lines, and the invisible portions are indicated with dashed lines. The regions involved in each interface are marked below the construct lines. (**F**) Binding free energy calculations based on MD–molecular mechanics/Poisson-Boltzmann surface area (MM/PBSA). The left panel shows the total binding free energy between the KIF3A/B tail and KAP3 (AB:K), and the individual contributions of KIF3A and KIF3B (A:K and B:K). The right panel summarizes the binding free energy contributions of each interface and the individual components of the complex. Bar graphs indicate means ± SD.

To understand how the KIF3 tail interacts with KAP3, we determined the high-resolution structure of the KIF3A/B/KAP3 cargo-binding domain (herein referred to as ABK) by collecting an extensive cryo-EM dataset. Data processing yielded two cryo-EM maps (2.83- and 2.75-Å resolution) (fig. S1 and table S1). The maps shared highly similar high-resolution regions, with class 1 containing an additional density. Thus, we selected the first map for de novo model building (*34*) and refinement (fig. S2). The final model resolved the core region of the ternary complex, encompassing KIF3A (residues 574 to 658), KIF3B (residues 580 to 675), and KAP3 ARM domain (residues 129 to 689) (Fig. 1, A to C). The reconstructed structure revealed four interaction interfaces between KIF3 and KAP3, including two tripartite interfaces involving both KIF3A and KIF3B (herein referred to as ABK interfaces 1 and 2) and two additional interfaces specific to KIF3B-KAP3 interactions (herein referred to as BK interfaces 1 and 2) (Fig. 1A). At the proximal tail region, adjacent to the stalk, both KIF3A and KIF3B form a helix–beta-hairpin–helix (herein referred to as H-βh-H) motif, which interacts with the C-terminal region of KAP3, constituting ABK interface 1 (Fig. 1, A to C). Further along the KIF3A/B tail, the extended loops reach toward both sides of KAP3, converging at ABK interface 2. Notably, the linking loop of KIF3A does not interact with KAP3 (Fig. 1B, arrow), whereas KIF3B engages in direct contact at BK interface 1 (Fig. 1, B and C). Subsequently, KIF3B extends further toward the N-terminal region of KAP3, forming BK interface 2. The residues at these binding interfaces are relatively well conserved, particularly at ABK interface 1 (Fig. 1D). Because of the inherent flexibility of certain regions, some segments were unresolved in the cryo-EM density map, preventing the modeling of KIF3A beyond ABK interface 2 (residues 658 to 701), KIF3B beyond BK interface 2 (residues 675 to 747), and the N-terminal segment of KAP3 (residues 1 to 129) (Fig. 1, C and E). The molecular dynamics (MD)–based binding free energy analysis revealed that KIF3A and KIF3B contribute ~28 and ~72%, respectively, to the overall interaction with KAP3 (Fig. 1F). Notably, KIF3A exhibits a stronger contribution at ABK interface 1, whereas its involvement in ABK interface 2 is relatively weaker (Fig. 1F).

**Fig. 2.**
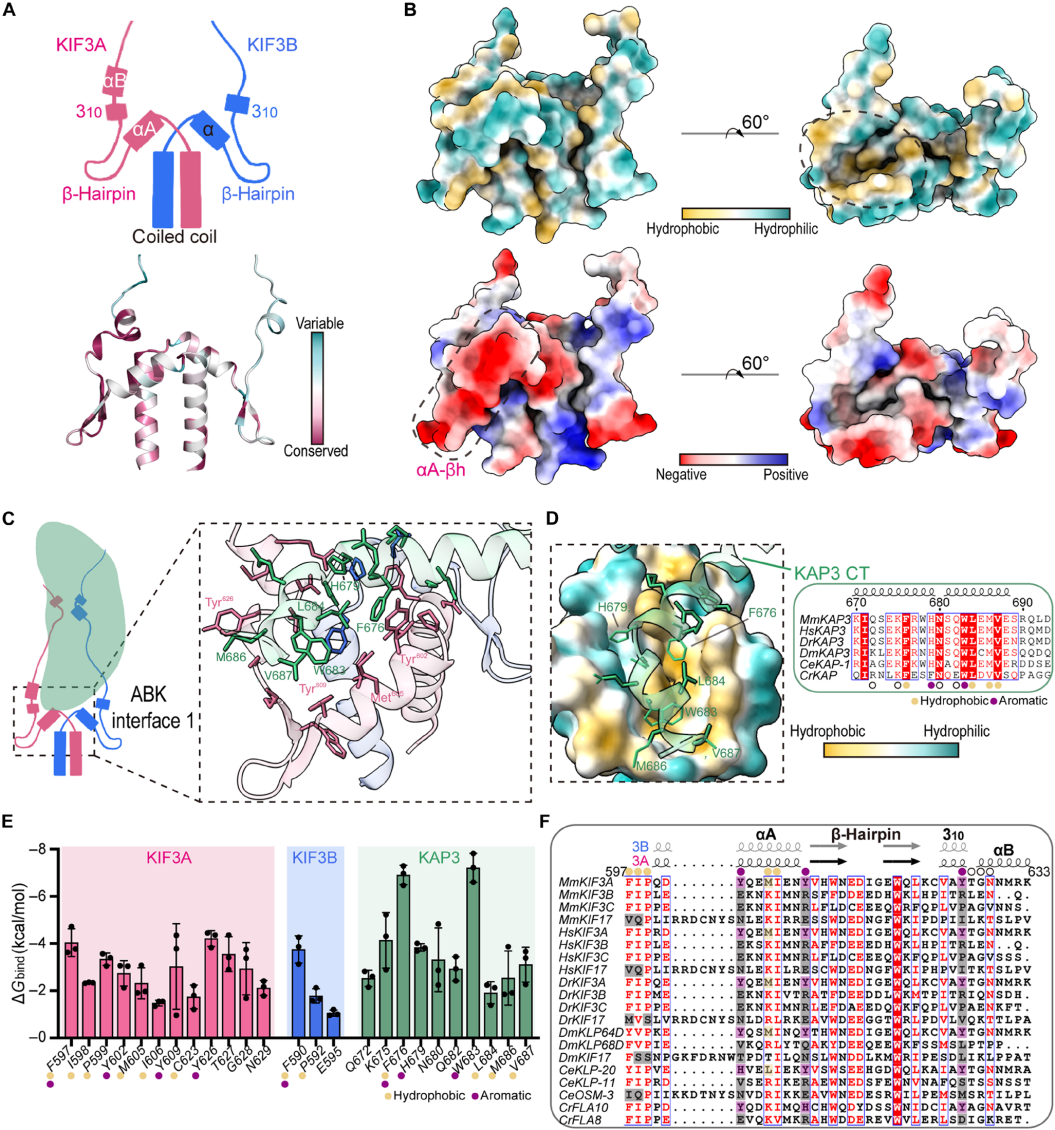
The stalk-tail junction of the KIF3 heterodimer forms a scaffold structure and binds KAP3. (**A**) Cartoon representation and conservation analysis of the stalk-tail junction of the KIF3 heterodimer. This region consists of a coiled-coil helix at the stalk terminus and an N-terminal H-βh-H motif in the tail. Conservation analysis reveals that KIF3A is more conserved than KIF3B in this region (bottom). (**B**) Surface hydrophobicity (top) and electrostatic properties (bottom) of the KIF3 stalk-tail junction. The dashed lines highlight the hydrophobic pocket (top right) and the negatively charged αA-βh region (bottom left), respectively. (**C**) Binding mode of the KIF3 stalk-junction region with KAP3 (ABK interface 1). The C-terminal helix of KAP3 binds to the hydrophobic pocket on the KIF3A side, with minor contributions from KIF3B. The zoom-in box is slightly rotated to better display the interacting residues. Conserved and important interacting residues are indicated. (**D**) Insertion of the KAP3 C-terminal helix into the hydrophobic pocket of the KIF3 scaffold (left) and sequence alignment analysis (right). Key hydrophobic and aromatic residues in KAP3 (e.g., Phe^676^, His^679^, Trp^683^, Leu^684^, Met^686^, and Val^687^) are highly conserved across species. Yellow circles mark hydrophobic residues, purple circles mark aromatic residues, and open circles mark residues involved in other interactions. (**E**) Binding free energy contributions of residues involved in ABK interface 1. Bar graphs indicate means ± SD. (**F**) Structure-based sequence alignment of KIF3 residues involved in the binding interface. Purple highlights indicate conserved residues in KIF3A critical for KAP3 binding, while gray highlights indicate nonconserved residues in KIF3B and KIF17.

Our structural model reveals a well-defined H-βh-H motif at the N-terminal tail region (Fig. 2A and fig. S3A). This motif consists of a shared α helix, β-hairpin, and 3_10_-helix (α-βh-3_10_) in both KIF3A and KIF3B. The C-terminal end of the KIF3 stalk engages with the H-βh-H motif, reinforcing structural stability (fig. S3A). Notably, in KIF3A, an additional short α-helix is observed following the 3_10_-helix, forming an αA-βh-3_10_-αB motif (Fig. 2A). This motif in KIF3A forms a hydrophobic pocket on one side (Fig. 2B, top right), while its αA-βh surface is highly enriched with negative charges (Fig. 2B, bottom left). Within ABK interface 1, the highly conserved C-terminal helix of KAP3 binds to this hydrophobic pocket (Fig. 2, C and D), primarily through hydrophobic and aromatic interactions with KIF3A (Fig. 2, D and E). Notably, β-hairpin regions do not participate in KAP3 binding.

The core residues forming the H-βh-H motif are highly conserved across the kinesin-2 family, including both heterodimeric (KIF3A/B and KIF3A/C) and homodimeric (KIF17) members (Fig. 2F), which suggests that the motif is a shared structural feature within kinesin-2 tails. However, the residues directly involved in KAP3 binding are not universally conserved. Specifically, the hydrophobic and aromatic residues critical for KAP3 binding in KIF3A (Tyr^602^, Met^605^, Tyr^609^, and Tyr^626^) are conserved within KIF3A homologs but not in KIF3B/C (kinesin-2β) or KIF17 (kinesin-2γ, KAP3 indispensable) (Fig. 2F). This suggests that KIF3A, the kinesin-2α subunit, plays a key role in KAP3 recognition.

### KIF3 tail establishes multiple binding sites with the concave surface of KAP3

**Fig. 3.**
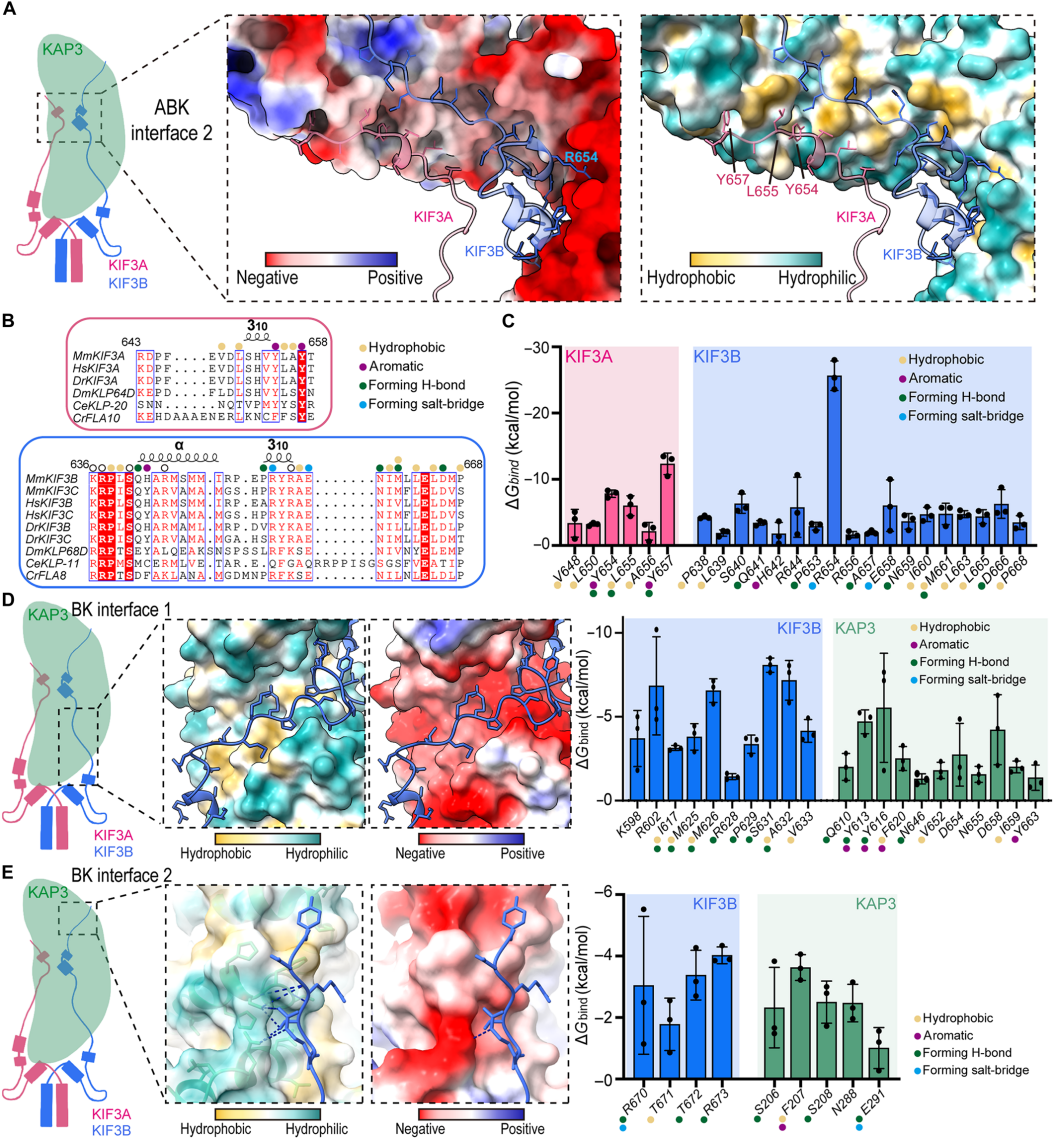
The KIF3 tail interacts with KAP3 at multiple sites. (**A**) ABK interface 2 on the concave surface of KAP3. The electrostatic (middle) and hydrophobic (right) properties of the KAP3 surface are shown, along with the cartoon structures of KIF3A/B segments involved in the interaction. Key interacting residues are labeled. (**B**) Sequence alignment of KIF3 residues involved in ABK interface 2. Short helices formed by KIF3 and key interacting residues are highlighted. (**C**) Binding free energy contributions of residues involved in ABK interface 2. Key interacting residues and interaction patterns are labeled. (**D**) First KIF3B-specific binding interface (BK interface 1). The electrostatic and hydrophobic properties of the KAP3 surface and the cartoon structure of the KIF3B segment are shown (left). Binding free energy contributions of residues involved in BK interface 1 are summarized (right). Key interacting residues are labeled. (**E**) Second KIF3B-specific binding interface (BK interface 2). The electrostatic and hydrophobic properties of the KAP3 surface, the cartoon structure of the KIF3B segment, and the hydrogen bond network are shown (left). Binding free energy contributions of residues involved in BK interface 2 are summarized (right). Key interacting residues are labeled. Bar graphs indicate means ± SD.

Following the formation of the ABK interface 1, the KIF3 tail extends further to establish a secondary binding interface, ABK interface 2, with the concave surface of the KAP3 mid-region (Fig. 3A). Unlike ABK interface 1, where KAP3 C-terminal helix fits into a hydrophobic pocket of KIF3, at ABK interface 2, both KIF3A and KIF3B adopt short helical conformations and interact with a highly conserved, electrostatically enriched, and partially hydrophobic concave surface of KAP3 (Fig. 3A and fig. S3, B and C). KIF3A engages in ABK interface 2 by forming a 3_10_-helix, anchoring to KAP3 with five hydrogen bonds (Fig. 3, B and C). Meanwhile, the proximal segment of KIF3B tail adopts both a 3_10_-helix and an α helix, where a cluster of seven positively charged residues (Arg^637^, His^642^, Arg^644^, Arg^654^, Arg^656^, Glu^658^, and Asp^666^) facilitates electrostatic interactions with the negatively charged surface of KAP3 (Fig. 3B). Further, the distal segment of the KIF3B tail interacts with the hydrophobic patch on KAP3 (Fig. 3A). Notably, KIF3B forms a total of 14 hydrogen bonds and two salt bridges with KAP3 (fig. S3, B and C), establishing an exceptionally stable binding interface. ABK interface 2 involves more extensive interactions (Fig. 3 and fig. S4), contributing approximately three times the binding energy compared to ABK interface 1 (Fig. 1F). Beyond ABK interfaces 1 and 2, KIF3B engages in two additional binding interfaces absent in KIF3A: BK interface 1, a 10-residue segment following the H-βh-H motif in KIF3B interacts with the dorsal surface of KAP3 (Fig. 3D); and BK interface 2, a five-residue extension from the distal KIF3B tail interacts with the N-terminal region of KAP3 (Fig. 3E). These additional binding sites contribute approximately one-fourth of the total binding energy (Fig. 1F), further reinforcing the stability of the ABK complex.

### APC binds to the core scaffold region of the KIF3 tail/KAP3 complex

**Fig. 4.**
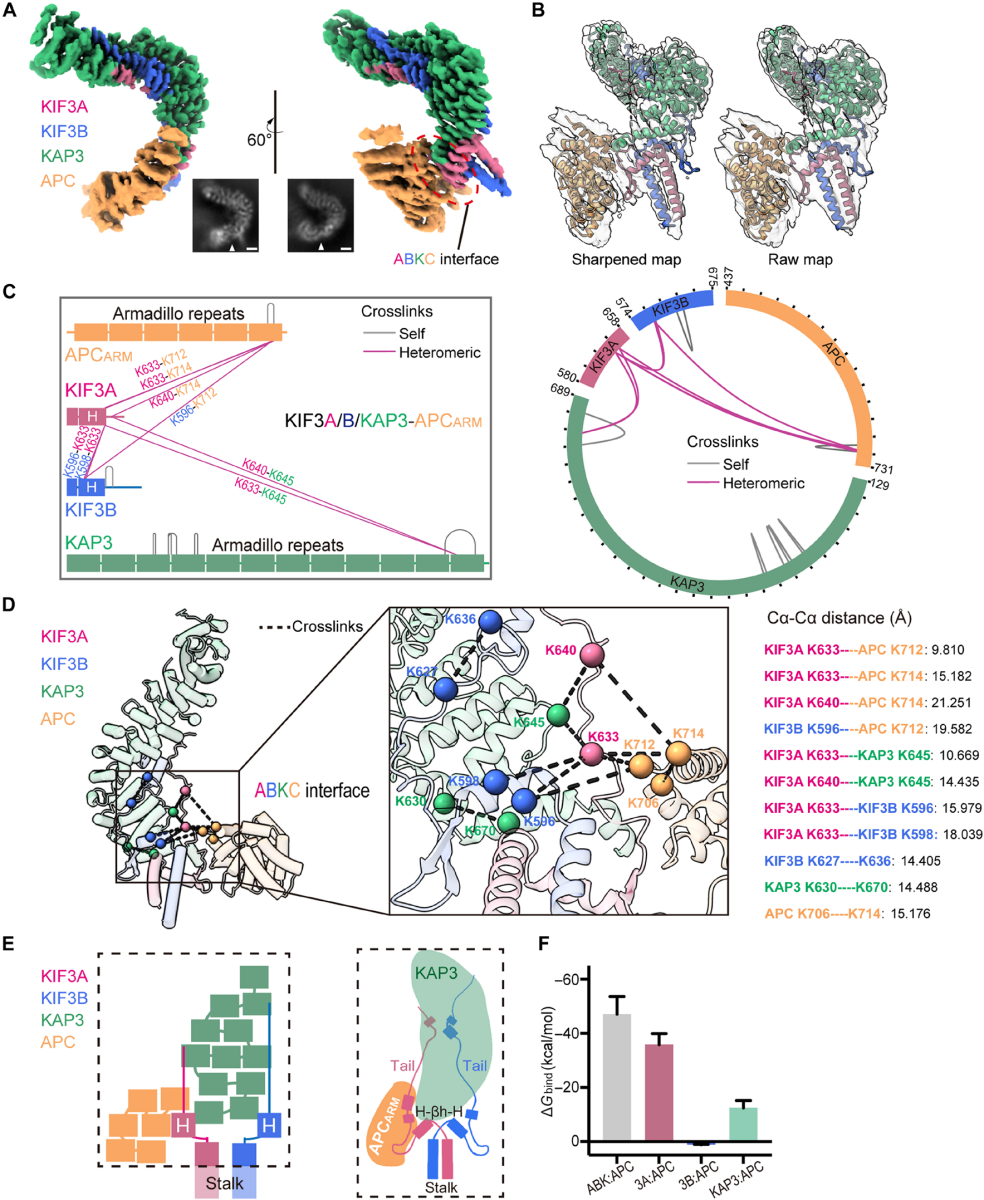
Structural determination and XL-MS analysis of the ABK-APC_ARM_ complex. (**A**) Cryo-EM map of the ABK-APC_ARM_ complex. The insets show representative 2D averages (The 2D averages are not exactly the same orientation as the 3D maps). The binding interface between the KIF3/KAP3 and APC_ARM_ is labeled (ABKC interface) on the map by a dashed circle and arrowheads on the 2D views. Rotations are shown to illustrate alternative orientations of the 3D reconstruction, whereas the 2D class averages represent views from approximate, but not strictly corresponding, angles. Scale bar for the 2D view, 2 nm. (**B**) Cryo-EM model of the ABK-APC_ARM_ complex. The cryo-EM map is shown in transparent gray. (**C**) 2D diagram (left) and circular representation (right) of XL-MS results in the ABK–APC_ARM_ model. Full data are available in fig. S7 and table S2. (**D**) XL-MS results mapped onto the structural model. Intermolecular cross-links are depicted using dashed lines in two colors on the left, each corresponding to the respective components. The KIF3A tail–linking loop region, including Lys^640^, was reintroduced from the initial docking model to allow full structural visualization. A focused view of the ABKC interface is presented in the center. Calculated distances between each cross-linked Cα atom pair are shown on the right, all within the permissible range of the BS3 cross-linker, supporting the structural integrity of the model. The KIF3 linking tail, not resolved in the ABK-APC EM map, is modeled here based on the aligned ABK structure to illustrate the cross-link between KIF3A Lys640 and its interacting partner. (**E**) Domain organization of the ABK-APC_ARM_ complex and a 2D diagram illustrating the binding mode. (**F**) Binding free energy calculations for ABK binding to APC_ARM_. The total binding free energy (ABK:APC) and individual contributions of KIF3A, KIF3B, and KAP3 (3A:APC, 3B:APC, and KAP3:APC) are shown. Bar graphs indicate means ± SD.

We next sought to understand how the ABK complex engages cargo proteins. To this end, we assembled a tetrameric KIF3A/B tail/KAP3-APC complex (*9*, *21*, *27*, *29*, *30*, *35*) using the validated KIF3/KAP3-binding ARM repeat region of APC and attempted single-particle cryo-EM reconstruction. The initial data collection was performed under experimental conditions similar to those used for ABK; however, the resulting reconstruction showed a severe orientation bias. To mitigate potential model-building errors arising from orientation bias, we collected an additional dataset using a tilted-stage setup with tilted angles set to 30° and 40° (fig. S5 and table S1). By merging particles from the two datasets, we improved the orientation distribution and ultimately obtained a higher-quality cryo-EM map and model (figs. S5 and S6). Compared to the KIF3/KAP3 map, the KIF3/KAP3-APC map revealed an additional density at the ABK interface 1 region, corresponding to the APC ARM domain (herein referred to as APC_ARM_) (Fig. 4A and fig. S5). The KIF3/KAP3 region in this map is highly consistent with the ABK map. The main differences include the absence of density for the highly flexible KIF3A tail–linking loop and the partial invisibility of the αB region within the KIF3A H-βh-H. In contrast, the coiled-coil stalk of KIF3A/B appears more extended in this map (Fig. 4, A and B, and fig. S6).

We docked the ABK structure, followed by integrating the available crystal structure of the APC_ARM_ [Protein Data Bank (PDB): 4YJE], into the sharpened map. We removed the KIF3A tail–linking loop region from the docked model and helical segments from the APC_ARM_ crystal structure that did not match the map. Ultimately, we successfully reconstructed the structure of the core ARM repeats of APC in complex with ABK. The model was refined using Phenix real-space refinement and Coot manual adjustments, improving its alignment with the EM density (Fig. 4B and fig. S6).

To further validate the model, we conducted cross-linking mass spectrometry (XL-MS) analysis (fig. S7 and table S2). We identified 15 lysine cross-links within our model—seven intramolecular and eight intermolecular (Fig. 4C and fig. S7). The KIF3A tail–linking loop region, including Lys^640^, was reintroduced from the initial docking model to allow full structural visualization (Fig. 4D and fig. S7B). All detected lysine pairs were within the spatial constraints of the BS3 cross-linker for Cα atom pairs, supporting the structural model, particularly the ABK-APC_ARM_–binding region (herein referred to as ABKC interface) (Fig. 4D). Based on the cryo-EM and XL-MS results, we found unexpectedly that the C-terminal ARM repeats of APC bind KIF3/KAP3 specifically at the ABK interface 1 region (Fig. 4E). As the αB region within the KIF3A H-βh-H domain was not fully built in the final map, we incorporated the KIF3A tail–linking loop region from the docking model for further MD-based free energy calculations. These calculations revealed that the KIF3A-APC_ARM_ interaction contributes the dominant binding energy—approximately three times greater than that of the KAP3-APC_ARM_ interaction—while KIF3B showed no contribution to APC_ARM_ binding (Fig. 4F).

### KIF3A and KAP3 cooperatively bind APC via a hydrophobic pocket

**Fig. 5.**
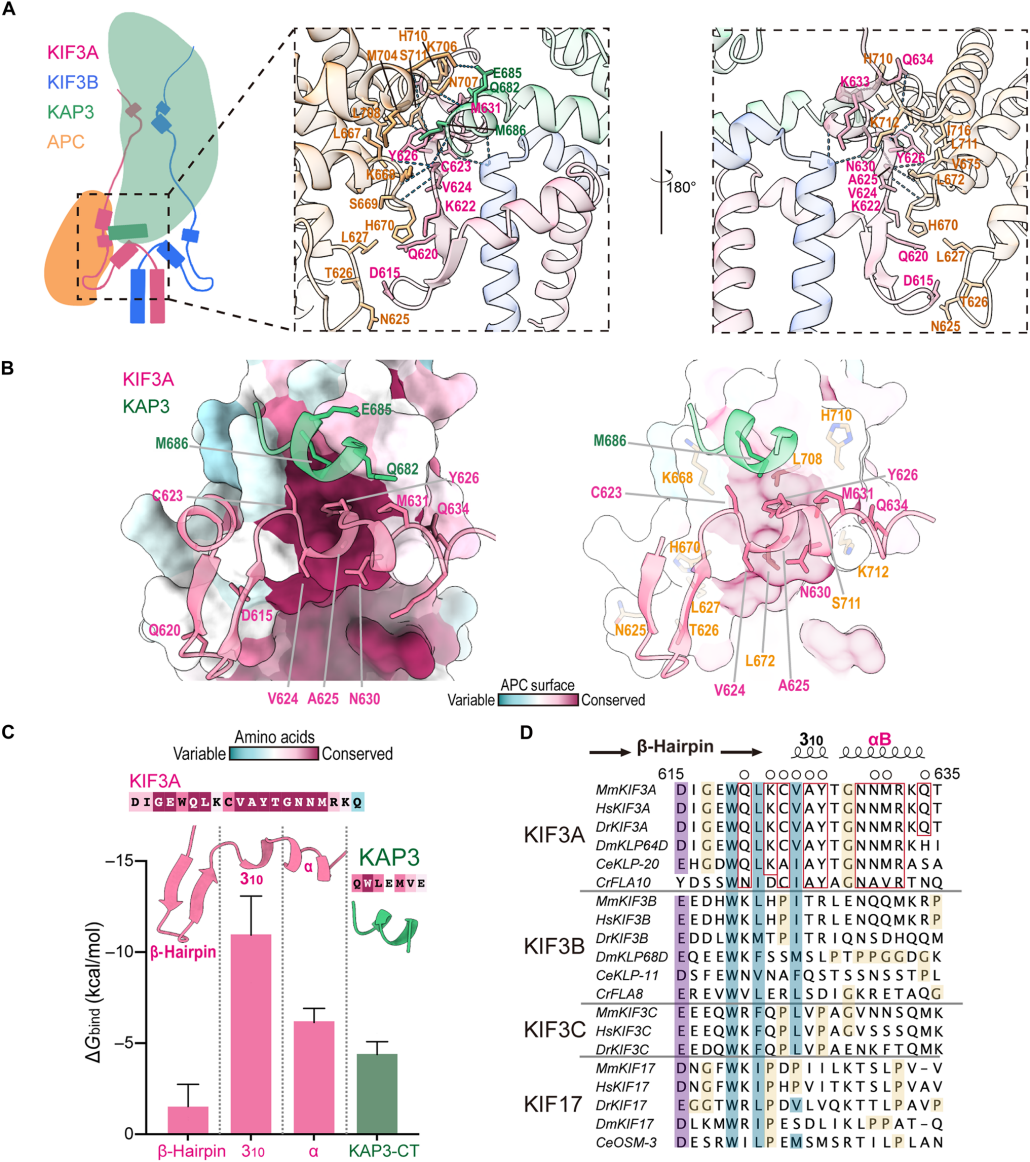
The KIF3A HAC domain is critical for specific APC_ARM_ binding. (**A**) Cartoon representation of the ABKC interface. The H-βh-H motif (HAC domain) of KIF3A and the C-terminal helix of KAP3 jointly bind the APC_ARM_. Key interacting residues and hydrogen bonds are labeled (right). (**B**) Conservation analysis of KIF3A/KAP3-binding surface of APC with interacting residues labeled. (**C**) Binding free energy and conservation analysis of the KIF3A/KAP3 interaction regions. (**D**) Sequence alignment of the KIF3A HAC domain across kinesin-2 family members. Purple highlights indicate conserved charged residues, cyan highlights indicate conserved hydrophobic/aromatic residues, and open circles mark residues involved in APC binding. Red boxes highlight KIF3A-specific conserved residues, and yellow highlights indicate proline residues in KIF3B/C and KIF17. The structure shown was derived from the MD simulation of our ABK-APC_ARM_ model after energy minimization and equilibration.

Because side-chain modeling for some residues in the ABKC region was not well resolved in our EM model, we used the energy-minimized and equilibrated structure from MD simulations for subsequent structural analysis. The ABKC interface primarily involves the βh-3_10_-αB segment within the H-βh-H of KIF3A, which contributes 11 residues, and the C-terminal helix of KAP3, which engages three residues, collectively interacting with 17 residues of the APC_ARM_ (Fig. 5A). These interactions are predominantly mediated by hydrophobic contacts and hydrogen bonding (fig. S8).

Within this interface, the 3_10_-αB region of KIF3A plays a central role, with Val^624^, Ala^625^, and Tyr^626^ from the 3_10_-helix inserting into a hydrophobic pocket formed at the C-terminal region of APC_ARM_. In addition, Cys^623^ and Asn^630^ contribute to stabilization through four hydrogen bonds with APC_ARM_ (Fig. 5A and fig. S8). Further interaction is mediated by Met^631^ and Gln^684^ from the αB helix, which establishes additional contacts with adjacent APC residues. Notably, these amino acids also play a crucial role in forming a hydrophobic pocket that interacts with the C-terminal helix of KAP3, and they contribute substantially to stabilizing this C-terminal helix of KAP3 through essential interactions (Fig. 2, C to F).

The KAP3 C-terminal helix also participates in the interaction of APC_ARM_, with Gln^682^ and Met^686^ engaging APC_ARM_. Notably, Met686 co-inserts into the hydrophobic pocket alongside the KIF3A 3_10_-helix, reinforcing the stability of the interface (Fig. 5A and fig. S8A).

In addition to these primary contacts, the β-hairpin region of KIF3A interacts with the central portion of the APC_ARM_ through Asp^615^ and Gln^620^ (fig. S8A). However, binding free energy calculations suggest that the interaction involving APC residues Asn^625^, Thr^626^, and Leu^627^ contributes minimally to the overall binding affinity (fig. S8B).

### The conserved helices within the KIF3A HAC motif dominate specific APC binding

Based on the functionally critical role of this H-βh-H motif in the binding of KAP3 adaptor and APC cargo and its structural characteristics as a protruding hook, we term it the kinesin-2 Hook-like adaptor and cargo-binding (HAC) domain (herein referred to as HAC domain) as it simultaneously functions to dock the adaptor and recognize the cargo. Conservation analysis reveals that the hydrophobic core within the APC_ARM_-binding pocket, where the KIF3A HAC domain interacts, is highly conserved (Fig. 5B). The APC_ARM_ interaction interface is enriched with hydrophobic residues, including multiple Leu, Ala, Ile, Val, and Met (fig. S8C). Similarly, the KIF3A HAC domain itself is highly conserved (Fig. 2A), with the helical region contributing most of the binding energy, while the β-hairpin provides only a minor contribution (Fig. 5C).

Despite a high degree of sequence similarity between KIF3B/C and KIF3A in the αA-β-hairpin region (Fig. 2F), the corresponding 3_10_-αB region of KIF3B/C exhibits substantial divergence from KIF3A (Fig. 5D). Within the kinesin-2β (KIF3B/C) subfamily, this region also shows relatively low conservation (Figs. 2A and 5D). Notably, many KIF3B/C and KIF17 homologs contain multiple proline residues in this region, which can disrupt helical formation and potentially alter the structural conformation (Fig. 5D).

**Fig. 6.**
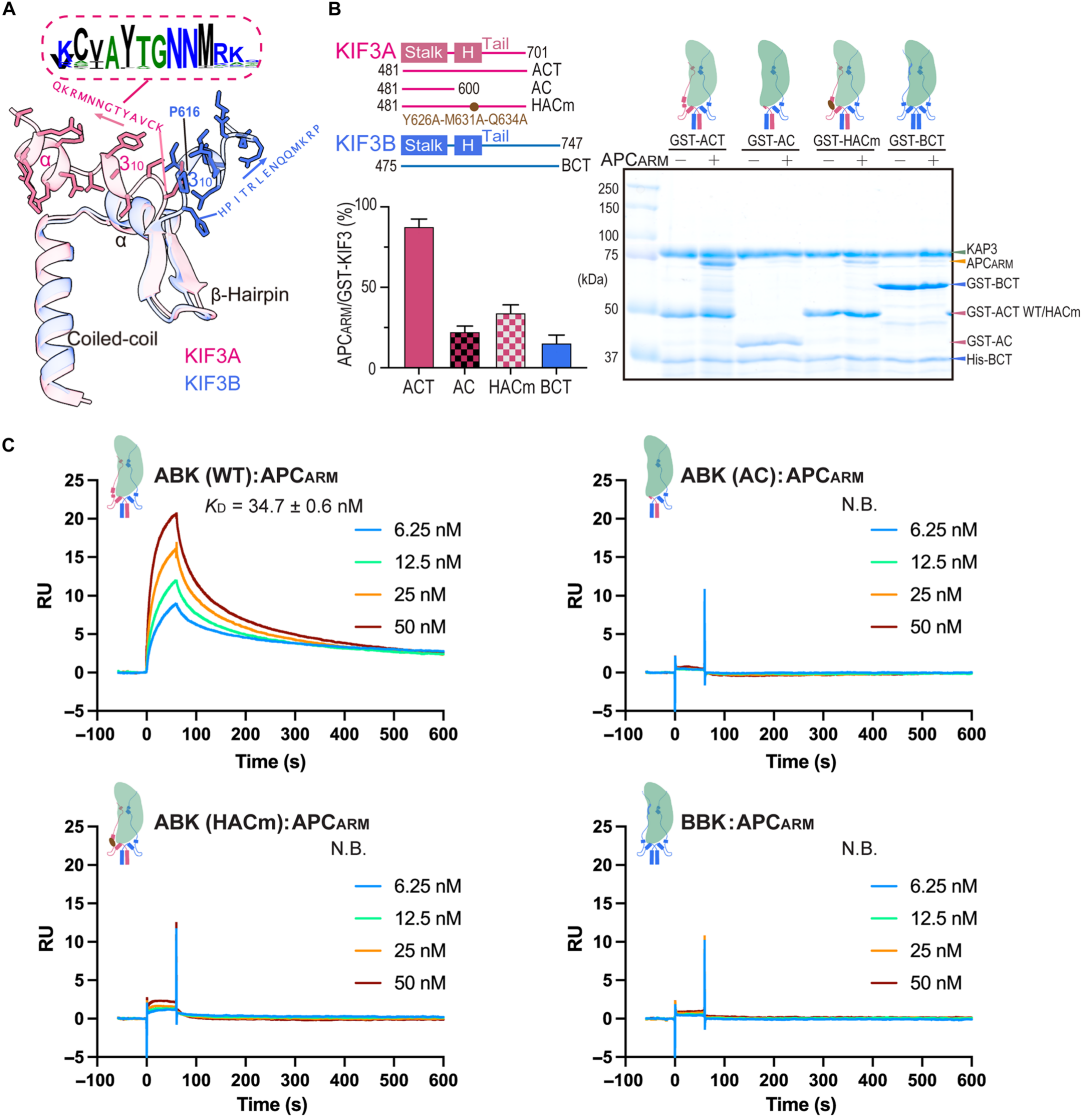
Biochemical evidence for the specific binding of APC_ARM_ to the KIF3A HAC region. (**A**) Structural alignment of the HAC motifs in KIF3A and KIF3B. (**B**) Pull-down analysis of the KIF3A HAC motif in APC binding. The upper left panel shows the KIF3 constructs, the lower left panel summarizes the results, and the right panel shows the Coomassie-stained SDS-PAGE gel. Bar graphs indicate means ± SD. (**C**) Quantitative binding analysis of APC_ARM_ interaction with kinesin-2 complexes by SPR. Shown are wild-type (WT) and mutant kinesin-2 complexes: ABK (WT), wild type; ABK (AC), KIF3A tail truncation; ABK (HACm), KIF3A Y626A-M631A-Q634A triple mutant; and BBK, KIF3B/B/KAP3 complex lacking KIF3A. RU, response units; *K*_D_, dissociation constant; N.B., no binding response; GST, glutathione *S*-transferase.

The structural alignment of the HAC regions in KIF3A and KIF3B reveals that the presence of Pro^616^ in KIF3B induces a loop reorientation following the β-hairpin and prevents the formation of the αB helix after the 3_10_-helix (Fig. 6A). In contrast, the 3_10_-αB region of KIF3A is highly conserved, forming a C/A-V/I-A-Y-T-G-N-N-M cargo recognition motif (Fig. 6A). Together with αA, this sequence motif shapes the hydrophobic pocket of the KIF3 HAC fold, which interacts with the KAP3 C-terminal helix and anchors the hydrophobic interface of APC_ARM_.

To explore the possibility of undetected interaction regions, we also performed AlphaFold3 structure predictions using the sequences of our constructs for three different complexes: AB (lacking KAP3 and APC_ARM_), ABK, and ABK-APC_ARM_. Compared to the AB alone, where the C-terminal tail of AB exhibited low confidence scores, the presence of KAP3 led to an increase in prediction confidence and revealed partial helical structures in the AB tail that interface with KAP3 (fig. S9). This is consistent with our cryo-EM ABK structure. Notably, a part of the N-terminal coiled-coil region present in the construct sequence, with high prediction confidence, was not observed in the EM density map, suggesting structural flexibility in this region under our experimental conditions (fig. S9). In addition, the prediction of the ABK-APC complex revealed that most regions of our APC construct outside the ARM repeats are flexible or helices of low confidence. These regions were not predicted to interact with ABK, consistent with our experimental observations where no corresponding density was resolved.

### Loss of APC_ARM_ binding upon disruption of the KIF3A HAC domain

To further validate these structural findings, we conducted biochemical pull-down assays using wild-type and mutated KIF3 C-terminal protein. Based on KIF3A C-terminal constructs (ACT), we generated a KIF3A tail truncation mutant (AC) and a triple alanine substitution mutant (HAC mutant, HACm: Y626A-M631A-Q634A) in the 3_10_-αB region (Fig. 6B). Pull-down assays showed that in the presence of KAP3 and KIF3B, wild-type ACT efficiently captured APC_ARM_ at an ~1:1 ratio. However, APC binding was markedly reduced in the truncated AC mutant, the HAC mutant, and the BCT construct lacking KIF3A (Fig. 6B). These results underscore the essential role of the KIF3A HAC site in APC_ARM_ binding. To address the limitations of the pull-down assay—such as potential nonspecific binding caused by excess APC_ARM_ protein and the use of glutathione *S*-transferase (GST) tags that may induce further oligomerization of kinesin-2 proteins—we further used surface plasmon resonance (SPR) to quantitatively assess the interaction. For this, we used His-tagged, size exclusion chromatography–purified wild-type and mutant kinesin-2 complexes. APC_ARM_ was immobilized at a constant level on a CM5 sensor chip, and binding affinities were quantitatively assessed. As shown in Fig. 6C, the wild-type kinesin-2 complex exhibited a strong binding affinity to APC_ARM_, with a dissociation constant (*K*_D_) of 34.7 ± 0.6 nM. In contrast, none of the three mutant kinesin-2 complexes—AC/ABK (with KIF3A tail truncation), HACm/ABK (with KIF3A Y626A-M631A-Q634A triple mutation), and BBK (lacking KIF3A)—displayed a detectable binding response under the same conditions (Fig. 6C). These results provide further biochemical validation of the structural model, highlighting the critical role of the KIF3A HAC domain in mediating the interaction between kinesin-2 and APC_ARM_.

### KIF3A HAC mutant disrupts APC dynamics and distribution in neurons

**Fig. 7.**
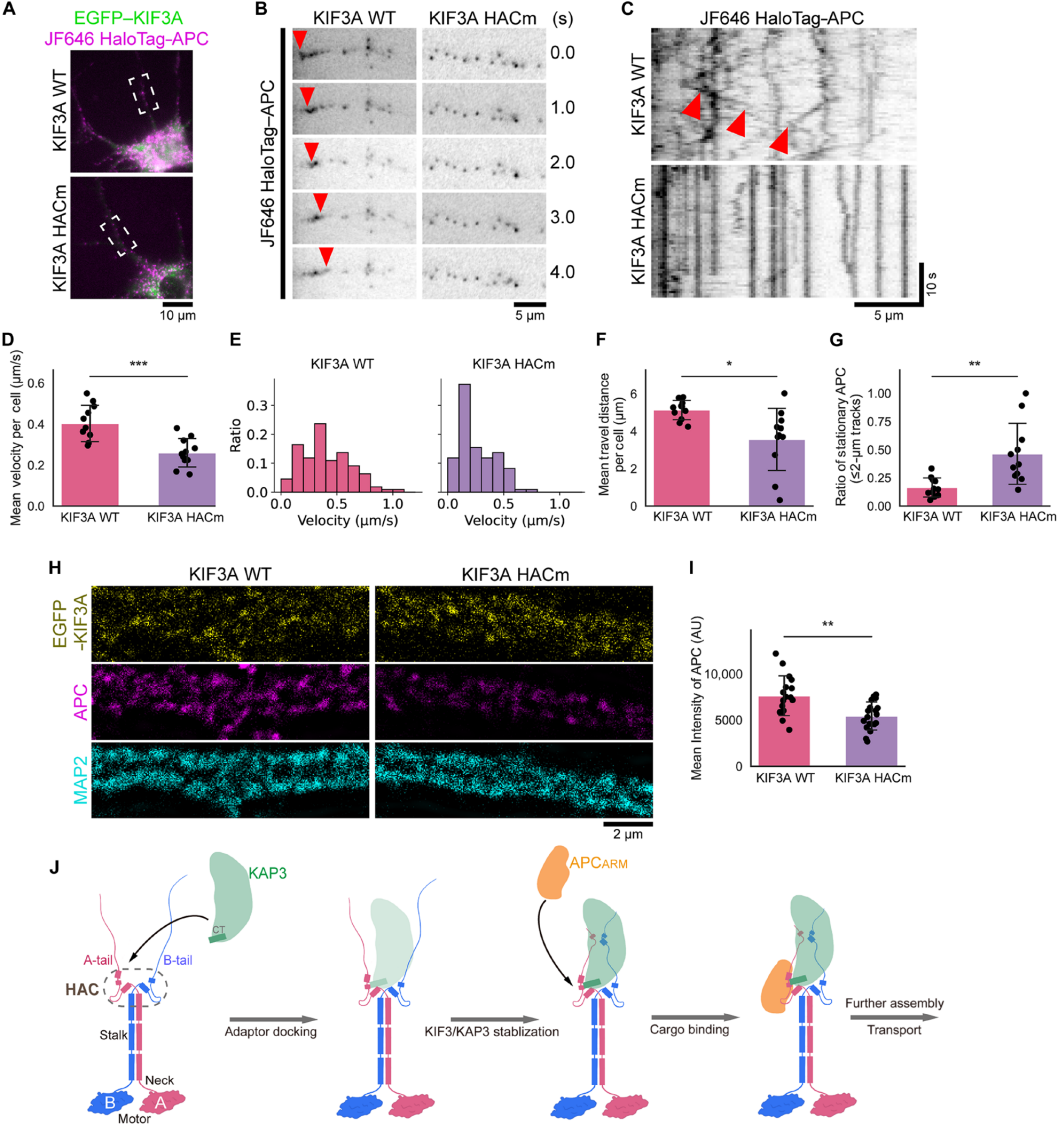
Disruption of APC transport and localization in primary neurons by KIF3A HAC mutant. (**A**) DIV9 (day in vitro 9) neurons coexpressing EGFP-KIF3A (WT or HAC mutant) and HaloTag-APC labeled with 1 nM JF646. The dashed box indicates the region shown in (B). (**B**) Time-lapse imaging of JF646 HaloTag-APC. Robust migration of APC was observed when coexpressed with KIF3A WT, whereas migration events were markedly reduced in the presence of the KIF3A HAC mutant. Arrowheads indicate representative migrating puncta. (**C**) Kymographs of JF646 HaloTag-APC. A higher number of stationary APC puncta were observed when coexpressed with the KIF3A HAC mutant. Arrowheads indicate representative migration events. (**D**) Mean velocity of APC per cell. (**E**) Histogram of velocities from 10 randomly selected tracks per neuron. *n* = 110 tracks from 11 neurons for each group. (**F**) Mean travel distance of APC per cell. (**G**) Ratio of stationary APC. Puncta with a travel distance ≤2 μm over 99.5 s were defined as stationary. (D), (F), and (G): Mean ± SD. *n* = 11 cells per group. *P < 0.05, **P < 0.01, and ***P < 0.001 by Welch’s *t* test. (**H**) Distribution of endogenous APC in DIV15 neurons transfected with KIF3A WT or HAC mutant. Fluorescence intensity along dendrites was reduced in KIF3A HACm-expressing neurons. (**I**) Quantification of endogenous APC intensity in dendrites. A significant reduction was observed in neurons expressing the KIF3A HAC mutant. Means ± SD. *n* = 17 (WT) and *n* = 18 (mutant) cells. **P < 0.01 by Welch’s *t* test. (**J**) Model of KIF3 binding to adaptor and cargo. First, the kinesin-2 dimer is formed through stalk-mediated dimerization, assembling the HAC domain in the tail; Second, HAC regions cooperatively recruit the KAP3 C-terminal helix, assembling the kinesin-2/adaptor complex. Third, the KIF3 tail further stabilizes KAP3 binding. Last, the KIF3A HAC domain specifically recognizes APC, working in concert with KAP3 to stably anchor and transport APC. HACm, KIF3A Y626A-M631A-Q634A triple mutant (KIF3A HAC mutant).

To investigate the effect of mutations in the KIF3A-APC interaction, we performed live imaging. Neurons were transfected with either wild-type KIF3A or its HAC mutant (Y626A-M631A-Q634A) together with full-length APC for visualization (Fig. 7A). As a result, robust dendritic migration of APC was observed when coexpressed with wild-type KIF3A, whereas APC migration was markedly reduced when coexpressed with the KIF3A HAC mutant (Fig. 7, B and C).

To statistically evaluate this effect, we quantified the mean velocity of APC in individual neurons, which revealed a significant reduction in APC velocity in the presence of KIF3A HAC mutant compared to KIF3A wild type (Fig. 7D). The histogram analysis of randomly selected APC puncta showed that slow-moving APC puncta with velocities less than 0.2 μm/s were more frequent under the HAC mutant condition (Fig. 7E). We then quantified the mean travel distance of APC puncta per cell and again found a significant reduction when coexpressed with KIF3A HAC mutant (Fig. 7F). Notably, the proportion of nearly stationary puncta (travel distance of ≤2 μm) was significantly increased in the KIF3A HAC mutant group (Fig. 7G).

Last, to examine whether these changes in migration dynamics affect APC distribution, we expressed either wild-type or mutant KIF3A alone in neurons and quantified endogenous APC. The fluorescence intensity of dendritic APC was significantly reduced in neurons expressing the KIF3A HAC mutant (Fig. 7, H and I). These results suggest that reduced binding affinity between the KIF3A mutant and APC impairs APC transport, resulting in decreased dendritic APC levels.

Integrating our structural, MD, biochemical, and neuronal analyses, we propose the following molecular model for heterodimeric kinesin-2 adaptor and cargo recognition (Fig. 7J). First, in dimerization and HAC assembly, the kinesin-2 dimer is formed through stalk-mediated dimerization, assembling the HAC domain in the tail. Second, in adaptor docking, the KIF3A/B HAC regions cooperatively recruit the KAP3, assembling the kinesin-2/adaptor complex, while the KAP3 C-terminal helix serves as an accessory component that further integrates the HAC domain. Third, in structural stabilization, the KIF3 tail region further stabilizes KAP3 binding, reinforcing the structural integrity of the complex. Last, in cargo recognition, the KIF3A HAC motif specifically recognizes APC cargo, working in concert with KAP3 to stably anchor the cargo (Fig. 7J).

## DISCUSSION

Motor proteins rely on adaptor proteins to ensure specific cargo recognition, transport regulation, and functional diversity (*4*, *5*). Our study provides the first high-resolution structural insights into the kinesin-adaptor-cargo binding, revealing a highly conserved H-βh-H motif in the KIF3 tail that serves as a central scaffold for KAP3 binding (Figs. 1 and 2). This motif, which we term the “HAC domain,” is critical for the formation of the primary binding interface, including between KIF3 and KAP3 (ABK interface 1), which stabilize the KIF3/KAP3 interaction through hydrophobic pockets, electrostatic interactions, and hydrogen bonding networks (Fig. 2). Notably, KIF3A plays a dominant role in APC binding, with its 3_10_-αB region engaging a unique hydrophobic pocket of APC_ARM_ (Figs. 4 and 5). In contrast, KIF3B contributes primarily to stabilizing the ABK complex through additional interfaces (ABK interface 2 and BK interfaces 1 and 2) (Fig. 3) but does not directly participate in APC recognition (Figs. 4 to 6). This functional divergence is likely due to structural constraints in KIF3B, such as the presence of proline residues that disrupt helical formation in its 3_10_-αB region (Figs. 5D and 6A). Our cryo-EM structural analysis was supported by both cross-linking data and AlphaFold3 predictions (Fig. 4 and figs. S7 and S9). Moreover, our findings were further validated by biochemical pull-down and SPR assays as well as cellular experiments using primary neuron cultures (Figs. 6 and 7). These results underscore the importance of the HAC region in kinesin-2 cargo recognition (Fig. 7J) and provide a structural basis for understanding how KIF3A and KAP3 cooperate to achieve specific cargo transport.

The discovery of the kinesin-2 HAC-KAP3 structure extends beyond kinesin-2, suggesting parallels and a similar structural framework among other molecular motors, such as kinesin-1 and dynein, which also use conserved hook-like structures for cargo recognition (fig. S10). Despite differences in composition, the recently resolved structure of the cytoplasmic dynein FHF adaptor complex exhibits a strikingly similar hook-like architecture to our ABK structure (*33*). The dynein/dynactin-associated HOOK protein contains a long coiled-coil stalk-like region, analogous to the stalk of kinesins, with its C-terminal coiled-coil and extension helices mediating interactions with both FHIPB and FTS (*32*, *33*, *36*). This interaction forms a complex highly reminiscent of the KIF3 tail/KAP3 structure analyzed in our study, probably serving as a tethering center for adaptors and cargo, same as the HAC region in kinesin-2 (fig. S10A). Moreover, the cargo-binding region formed by the C-terminal region of the kinesin-1 heavy chain and its light chain (KLC) is also proposed to adopt a hook-like structure (*8*). The tetratricopeptide repeat domain of KLC shares structural similarity with the ARM domain of KAP3, suggesting that their overall architecture may resemble the HAC-KAP3 structure (fig. S10A), although this awaits further validation. We speculate that other motor proteins may also form tethering centers, functional akin to the HAC region, at their tail ends or through primary adaptors, subsequently adopting hook-like architectures to facilitate coordinated interactions with adaptors and cargo. We also propose a unified model for cargo recognition in kinesin and dynein. The C terminus of adaptors in kinesin 1/2 and dynein features a helix-based adaptor/cargo tethering site that promotes adaptor assembly and facilitates subsequent cargo recognition and binding (fig. S10B). The role of the tethering site in cargo binding may vary: For kinesin-1 and dynein, cargo specificity arises from the interaction between cargo and adaptor proteins, while in kinesin-2, cargo specificity is determined by the tethering site itself.

Nevertheless, although cargo-binding units of kinesin-1 and dynein exhibit some structural similarities with kinesin-2, they share no sequence homology. This suggests that any shared cargo-binding mechanism, if present, may exist only at a macroscopic or architectural level. The unique HAC structure may play a critical role in defining the functional specificity of kinesin-2. Beyond its role in neuronal transport, the most well-known function of kinesin-2 is its mediation of anterograde IFT. Recent studies have shown that kinesin-2 interacts with the IFT-B complex, possibly at the junction between the B1 and B2 subcomplexes (*37*). However, structural evidence supporting this interaction remains lacking. The identification of a conserved cargo recognition motif (C/A-V/I-A-Y-T-G-N-N-M) within the KIF3A HAC domain, along with the corresponding conserved KIF3A-binding pocket in APC, has notable practical implications. These findings could inform future investigations into other KIF3-specific cargos, particularly those involving the IFT complex, expanding our understanding of the kinesin-2–mediated transport mechanism.

Furthermore, the HAC region contains phosphorylatable residues [e.g., Tyr^626^ (*38*)], which may serve as regulatory sites to modulate KIF3 binding to APC or other cargoes. The Tyr^626^ is followed by threonine (T) and glycine (G), forming a YTG motif that is typically recognized by Src family kinases. The presence of glycine contributes to local flexibility, which may facilitate phosphorylation by Src kinases. The upstream residues WQLK (tryptophan, glutamine, leucine, and lysine) provide a combination of hydrophobic and charged side chains, which may enhance binding affinity to the SH2 or SH3 domains of Src family kinases. Now, kinesin-2 is known to be regulated by the serine/threonine kinases intestinal cell kinase (ICK), male germ cell associated kinase (MAK), protein kinase A (PKA), and calcium/calmodulin-dependent protein kinase type II alpha (CaMKIIa), which modulates IFT (*39*) or neuronal transport (*24*). However, no tyrosine kinase–mediated regulatory pathway has been reported to date. As a next step, it would be worthwhile to screen for potential upstream tyrosine kinases that may regulate ciliary or neuronal function through phosphorylation of kinesin-2. These posttranslational modifications might also integrate kinesin-2 function into broader cellular signaling networks, such as the Wnt/β-Catenin pathway (*40*). However, certain limitations remain for this study. For example, the high flexibility of some regions in the KIF3 tail and KAP3 prevented their high-resolution analysis in our cryo-EM maps (Fig. 1E), leaving open the possibility of additional binding or regulatory sites. Future studies could use advanced structural techniques or biochemical assays to explore these unresolved regions and investigate the dynamic regulatory mechanisms of the HAC region, such as phosphorylation-dependent modulation of cargo binding.

## MATERIALS AND METHODS

### Constructs, protein expression and purification

The gene encoding the C-terminal region of *Mus musculus* KIF3A [National Center for Biotechnology Information (NCBI) accession: NP_032469.2; residues 481 to 701, ACT) was cloned into a pETDuet-1 vector with an N-terminal 6His tag, while the C-terminal region of *M. musculus* KIF3B (NCBI accession: NP_032470.3; residues 475 to 747, BCT) inserted into another multiple cloning site without expression tags for the coexpression of the KIF3A/B tail heterodimer. These sequences were also cloned into a pGEX-6p-3 vector containing an N-terminal GST tag for pull-down assays. A truncated form of KIF3A (AC, residues 481 to 600) and a triple point-mutant variant (HAC mutant) were generated using polymerase chain reaction (PCR) for both the pGEX-6p-3 vector and pETDuet-1 vector.

*M. musculus* KAP3A (residues 1 to 693, NCBI accession: NP_001292572.1) and the ARM domain of *M. musculus* APC (NCBI accession: AAB59632.1; residues 338 to 1010) were PCR-amplified and cloned into a pET-21b vector without expression tags. Recombinant plasmids were transformed into *Escherichia coli* BL21 (DE3) (Novagen), and both ABK and ABK-APC_ARM_ complexes were reconstituted and purified as previously described (*9*). Purified proteins were concentrated to 10 mg/ml using ultrafiltration, flash-frozen in liquid nitrogen, and stored at −80°C.

### Cryo-EM sample preparation and data collection

For cryo-EM analysis of both ABK and ABK-APC_ARM_ complexes, 3 μl of the sample at a concentration of 0.75 mg/ml was applied onto a hydrophilized holey carbon grid (Cu, R1.2/1.3, 300 mesh, Quantifoil). The grid was then blotted for 4 s (blot force 10) and plunge-frozen in liquid ethane using a Vitrobot Mark IV (FEI) at 6°C and 100% humidity.

Images were acquired on a CRYO ARM 200 (JEOL) microscope operating at 200 kV using SerialEM for automated data collection. Movie frames were recorded with a Gatan K3 direct electron detector operated in counting mode and correlated-double sampling (CDS) mode at a nominal magnification of ×80,000, yielding a pixel size of 0.571 Å/pixel. The data were collected in zero-loss mode with an energy filter slit width of 20 eV and an objective lens aperture diameter of 150 μm. For the ABK sample, 34,176 movies were recorded with a total electron exposure of 65.3 e^−^/Å^2^ over 75 frames. For the ABK-APC_ARM_ sample, 36,020 movies were recorded with a total electron exposure of 64.7 e^−^/Å^2^ over 80 frames. The defocus range was set from −0.6 to −1.8 μm in 0.1-μm increments. A summary of the imaging parameters is provided in table S1.

### Cryo-EM tilted-stage data collection

For tilted-stage data collection, 3 μl of ABK-APC_ARM_ at a concentration of 0.375 mg/ml was applied onto a hydrophilized holey Au grid (UltrAufoil R1.2/1.3, 300 mesh, Quantifoil). The grid was then blotted for 4 s (blot force 10) and plunge-frozen in liquid ethane using a Vitrobot Mark IV (FEI) at 6°C and 100% humidity.

Images were acquired on a Titan Krios G3i (Thermo Fisher Scientific) microscope operating at 300 kV using EPU software (Thermo Fisher Scientific) for automated data collection. Movie frames were recorded with a Gatan K3 direct electron detector operated in counting mode and CDS mode at a nominal magnification of 130,000×, yielding a pixel size of 0.65 Å/pixel. The data were collected in zero-loss mode with an energy filter slit width of 20 eV and an objective lens aperture diameter of 100 μm; tilted angle was set to 30° and 40°, each applied to half of the grid squares. Movies (19,918) were recorded with a total electron exposure of 63 e^−^/Å^2^ over 63 frames. The defocus range was set from −1.0 to −1.6 μm in 0.2-μm increments. A summary of the imaging parameters is provided in table S1.

### Cryo-EM single-particle data processing

All data were processed using CryoSPARC (version 4.6.2) (*41*). First, raw movies were subjected to Patch Motion Correction to compensate for beam-induced sample drift, followed by Patch CTF Estimation to determine contrast transfer function (CTF) parameters. Dose-weighted images with a CTF fit resolution worse than 8 Å were discarded, leaving 33,576 and 35,534 images for the ABK and ABK-APC_ARM_ datasets, respectively, for further particle picking. For particle selection, an initial Blob Picker job was used to generate preliminary two-dimensional (2D) templates. These templates were refined through 2D classification, and the improved templates were then used for Template Picker to enhance the accuracy of particle selection. To mitigate orientation bias, multiple rounds of template picking were conducted with varying particle sizes to ensure diverse angular coverage. Extracted particles were down-sampled to a pixel size of 2.284 Å (4× binning) with a box size of 228 Å for initial processing. Duplicates from multiple rounds of template picking were merged and removed. Particles were classified into 300 classes, and poorly defined classes were discarded. Several rounds of 2D classification were performed using different mask sizes (90, 110, 130, 150, and 170 Å) to capture particles with a broader range of orientations. Well-defined 2D classes from these classifications were selected, merged, and deduplicated. This resulted in 9,701,817 particles for ABK and 6,456,283 particles for ABK-APC_ARM_, which were used for 3D reconstruction.

A subset of particles was used to generate six initial 3D models using ab initio reconstruction. These models were then used in heterogeneous refinement to classify particles into distinct conformational or compositional states. To improve data quality, selected 3D classes were re-extracted at a pixel size of 1.142 Å (2× binning) and underwent multiple rounds of ab initio reconstruction and heterogeneous refinement to discard low-quality particles. For ABK, two well-defined classes with good orientation distributions were selected and refined separately using nonuniform refinement (*42*) to improve map quality and resolution.

In contrast, the ABK-APC_ARM_ dataset exhibited a severe orientation bias. To address this, dominant-view particles were down-sampled using the Rebalance Orientation tool. A total of 615,384 particles were extracted at a pixel size of 1.29994 Å and combined with 570,175 particles from a tilted-stage dataset, which were processed similarly at a pixel size of 1.3 Å. Heterogeneous refinement and nonuniform refinement were performed on the combined dataset. The Exposure Group Utilities job was used to define optical groups, followed by further CTF refinement and local refinement. Last, 3D classification without alignment was performed. The best class, containing 407,130 particles, was selected for final local refinement. As a result, the orientation bias was substantially reduced, and the overall map quality was markedly improved.

Local resolution estimation was conducted to assess the local resolution distribution. The final map was sharpened using DeepEMhancer (*43*). A summary of the data processing workflow and final reconstruction resolutions is provided in fig. S1 for ABK and fig. S5 for ABK-APC_ARM_. The statistics were summarized in table S1.

### Model building and validation

For ABK, the class 1 sharpened map was used for de novo model building with ModelAngelo (*34*) to generate an initial model. The raw map was used to supplement the construction of the linking loop in the KIF3A tail region. The model was then imported into Phenix (*44*) for automated real-space refinement, followed by manual corrections using Coot (*45*).

For the ABK-APC_ARM_, the initial model was constructed by docking the ABK model solved in this study and integrating the available crystal structure of the APC_ARM_ domain (PDB: 4YJE). The model was further refined using Phenix, followed by manual adjustments in Coot to improve alignment with the EM density. The overall model-building workflow is illustrated in fig. S2 (for ABK) and fig. S6 (for ABK-APC_ARM_).

Model validation was performed using MolProbity (*46*) to assess stereochemical quality. A summary of model-building and refinement statistics is provided in table S1.

### MD simulation and free energy calculation

The MD simulations of ABK and ABK-APC_ARM_ were performed using GROMACS (version 2024.4), with slight modifications to the protocol previously described (*39*). Briefly, missing side chains in the low-resolution region were modeled through UCSF ChimeraX, and the topology files were generated using the OPLS-AA force field parameter set. Each system was solvated in 150 mM NaCl with simple point charge-extended water models in a cubic box. Neutralizing counterions were added, and the steepest descent energy minimization was performed. This was followed by a two-step equilibration: 100 ps of isochoric-isothermal equilibration and 100 ps of isothermal-isobaric equilibration. All position restraints were removed, and simulations were run for 1 ns. Root mean square deviation (RMSD) analyses were used to calculate the standard deviation (SD) of atomic positions for specified amino acids compared to their initial positions within the energy-minimized and equilibrated structures. Each simulation was performed in triplicate.

Based on the RMSD stability observed during the 1-ns MD simulation, we selected 20 stable frames from the 0.8- to 1-ns interval to estimate the binding free energy between the proteins using the gmx_MMPBSA tool (*47*). The molecular mechanics/Poisson-Boltzmann surface area (MM/PBSA) method was applied, partitioning the free energy into molecular mechanical energies (electrostatic and van der Waals), polar solvation energies (calculated via the Poisson-Boltzmann equation), and nonpolar solvation energies (estimated from the solvent-accessible surface area). To identify key residues contributing to binding, per-residue energy decomposition was performed using the decomposition feature of gmx_MMPBSA. The data of total delta free energy were plotted using GraphPad Prism7 (RRID: SCR_002798).

### Cross-linking mass spectrometry

To investigate potential interactions among the KIF3A/B tail, KAP3, and APC_ARM_, ABK-APC_ARM_ complex was cross-linked using bis(sulfosuccinimidyl) suberate (BS3; Dojindo) for 10 min at room temperature, followed by quenching with 50 mM tris-HCl. The sample was then digested with trypsin, desalted, and analyzed using a ZenoTOF7600 mass spectrometer (SCIEX) coupled with an UltiMate3000 RSLCnano system (Thermo Fisher Scientific). Cross-linked fragments containing BS3 were analyzed using MaxLynx within the MaxQuant software suite (*48*). A summary of detected cross-links is provided in table S2.

### Conservation analysis

To evaluate the evolutionary conservation of the proteins KIF3A, KIF3B, KAP3, and APC_ARM_ within our structural models, we used the ConSurf server (*49*) to analyze the phylogenetic relationships among homologous sequences to determine the conservation levels of amino acid positions. The amino acid sequences of KIF3A, KIF3B, KAP3, and APC_ARM_ were submitted to ConSurf, which automatically identified homologous sequences using the HMMER algorithm against the UniRef90 database, applying an *E* value cutoff of 0.0001 to ensure robust matches. Redundant sequences were filtered out, and the remaining sequences were aligned using the MAFFT-L-INS-i algorithm for multiple sequence alignment (MSA). A phylogenetic tree was constructed from the MSA using the neighbor-joining method. Subsequently, the Rate4Site algorithm calculated evolutionary conservation scores for each amino acid position, assigning grades from 1 (most variable) to 9 (most conserved) based on evolutionary rates. These conservation scores were then mapped onto the 3D structures of the proteins, facilitating visualization of conserved and variable regions.

### Sequence alignment, AlphaFold multimer prediction, and structural analysis

Structure-based MSAs were performed using Clustal Omega (RRID: SCR_001591) and ESPript server (RRID: SCR_006587). The protein sequences used for analysis were obtained via the NCBI protein database under the accession numbers NP_001287720.1 (*Homo sapiens* KIF3A), NP_001277734.1 (*M. musculus* KIF3A), NP_001093615.1, (*M. musculus* KIF3B), NP_001093615.1, NP_001093615.1 (*Danio rerio* KIF3A), (*D. rerio* KIF3B), NP_523934.1 (*Drosophila* Klp64D), NP_497178.1 (*Caenorhabditis elegans* Klp-20), XP_001701510.1 (*Chlamydomonas* FLA10), NP_004789.1 (*H. sapiens* KIF3B), NP_032470.3 (*M. musculus* KIF3B), NP_001093615.1 (*D. rerio* KIF3B), NP_524029.2 (*Drosophila* Klp64D), NP_741473.1 (*C. elegans* Klp-11), and XP_001697037.1 (*Chlamydomonas* FLA8), NP_002245.4 (*H. sapiens* KIF3C), NP_032471.2 (*M. musculus* KIF3C), XP_002661420.3 (*D. rerio* KIF3C), NP_065867.2 (*H. sapiens* KIF17), NP_034753.1 (*M. musculus* KIF17), XP_068080281.1 (*D. rerio* KIF17), NP_651939.4 (*Drosophila* KIF17), NP_001367796.1 (*C. elegans* OSM-3), NP_001191443.1 (*H. sapiens* KAP3), NP_035288.2 (*M. musculus* KAP3), NP_001004644.1 (*D. rerio* KAP3), NP_001138186.1 (*Drosophila* KAP3), NP_001021247.1 (*C. elegans* KAP-1), XP_001698323.1 (*Chlamydomonas* KAP), NP_000029.2 (*H. sapiens* APC) NP_001389660.1 (*M. musculus* APC), NP_001137312.1 (*D. rerio* APC), and NP_001263046.1 (*Drosophila* APC).

The AlphaFold Multimer structural prediction of kinesin-2 complexes was performed using AlphaFold 3 (https://alphafoldserver.com) (*50*). Structural analysis was performed, and structural figures were generated using the program UCSF ChimeraX (RRID: SCR_015872).

### Pull-down assay

For the pull-down analysis using wild-type KIF3A/B tail and mutated KIF3A tail, *Escherichia coli* cells expressing the bait proteins (GST-fused C-terminal KIF3A or KIF3B constructs) (Fig. 6B) were premixed with *E. coli* cells overexpressing input proteins (KIF3B tail, KAP3, and APC_ARM_). The mixtures were lysed by sonication in buffer containing 20 mM tris-HCl (pH 8.0), 150 mM NaCl, 7 mM β-mercaptoethanol, and 5% (v/v) glycerol.

After centrifugation, the cleared supernatants were incubated with pre-equilibrated Glutathione Sepharose 4B beads (Cytiva) for 1 hour at 4°C on a rocker. The bound proteins were eluted with 10 mM reduced glutathione and analyzed by SDS-PAGE followed by Coomassie Brilliant Blue staining. All experiments were performed in triplicate. Quantification was performed using ImageJ (RRID: SCR_003070), and statistical analysis was conducted with GraphPad Prism7 (RRID: SCR_002798).

### Surface plasmon resonance

SPR analysis was performed using a Biacore T200 instrument (Cytiva, Marlborough, MA, USA) to assess the binding affinities between wild-type or mutant kinesin-2 complexes and APC_ARM_. APC_ARM_ was immobilized on a CM5 sensor chip (Cytiva) at around 200 response units (RU) using standard amine coupling chemistry in an immobilization buffer containing 10 mM sodium acetate (pH 5.5). The running buffer consisted of 20 mM Hepes (pH 8.0), 150 mM NaCl, 1 mM dithiothreitol, and 0.005% Tween 20. Kinesin-2 proteins were injected at concentrations ranging from 6.25 to 50 nM at a flow rate of 30 μl/min, with an association phase of 60 s and a dissociation phase of 540 s at 15°C. Sensorgrams were analyzed using BIAevaluation software (version 3.2.1, Cytiva), and equilibrium dissociation constants (*K*_D_) were calculated on the basis of Scatchard plot analysis.

### Cell culture, imaging, and quantification

Hippocampi were dissected from ICR mice (Charles River Laboratories; IMSR catalog no. CRL:022, RRID: IMSR_CRL:022) on embryonic day 16. No gender determination was done, and three embryos were used. The hippocampi were digested with 0.25% trypsin (Thermo Fisher Scientific) in Hanks’ balanced salt solution (FUJIFILM Wako) for 15 min at 37°C. Dissociated hippocampal cells were seeded at a density of 3.5 × 10^4^ cells per well on eight-well chamber cover (Matsunami Glass) coated with 0.04% polyethylenimine (Merck) and BioCoat poly-d-lysine (Corning). Neurons were cultured in minimum essential medium (Thermo Fisher Scientific) supplemented with 1 mM pyruvate (Thermo Fisher Scientific), 0.6% glucose, 2 mM GlutaMAX (Thermo Fisher Scientific), 2% B27 Plus (Thermo Fisher Scientific), and penicillin-streptomycin (100 U/ml; Thermo Fisher Scientific). The cells were maintained at 37°C in a humidified atmosphere of 95% air and 5% CO_2_. Cultured neurons were transfected with the High-Efficiency Ca^2+^ Phosphate Transfection Kit (Takara Bio) according to the manufacturer’s protocols. For EGFP-KIF3A alone, 0.5 μg of plasmid DNA was transfected per well at 14 days in vitro (DIV14). For cotransfection, 0.25 μg each of EGFP-KIF3A and HaloTag-APC plasmids (Promega, #FHC01190) was transfected per well at DIV9. These experiments have passed a rigorous ethical review and have been approved by Gunma University for animal experiments (approval number 24-056) and genetic recombination experiments (approval number 24-060).

For live imaging, HaloTag-APC was incubated with 1 nM JF646 HaloTag ligand (Promega) for 15 min, followed by two quick washes with culture medium. Live imaging was performed 8 to 10 hours after transfection using a Nikon Eclipse Ti inverted microscope. Excitation was achieved with a 488-nm sapphire laser (Coherent) and a 647-nm fiber laser (MPB Communications), and imaging was conducted using a Plan Apo TIRF 100× oil immersion objective with the incident angle adjusted to achieve HILO illumination. The focal plane was maintained using the perfect focus system (PFS), and imaging was carried out in a 5% CO_2_ at 37°C incubator (BLAST Co., Kawasaki, Japan). Time-lapse images were acquired using an electron-multiplying charge-coupled device camera (iXon3 DU897, Oxford Instruments) at a resolution of 512 × 512 pixels, with an exposure time of 0.5 s and a gain of 300. KIF3A was imaged only in the first frame, whereas APC was recorded continuously for 200 frames over 99.5 s.

Live imaging data were first drift-corrected using the Correct 3D Drift plugin in ImageJ and subsequently analyzed with the TrackMate plugin. A 20-μm region of interest (ROI) at the proximal dendrites of each neuron was defined. Moving puncta were detected using the Laplacian of Gaussian detector with an estimated blob diameter of 0.6 μm and a threshold to exclude weak signals. Trajectories were then generated using the Kalman tracker with a search radius of 1.2 μm and a maximum frame gap of 2. Tracks shorter than 10 frames were excluded from analysis. Among the output features, TRACK_MEAN_SPEED and TOTAL_DISTANCE_TRAVELED were subjected to statistical analysis.

Immunostaining was performed 24 hours after transfection. Neurons were fixed with 4% paraformaldehyde, permeabilized with 0.5% Triton X-100, and blocked with 5% bovine serum albumin. Primary antibodies [anti-APC (Santa Cruz Biotechnology, catalog no. sc-896, RRID: AB_2057493) and anti-MAP2 (Novus, catalog no. NB 300-213, RRID: AB_350528)] were applied at room temperature for 1 hour. After washing with PBS for 3× 5 min, cells were incubated with secondary antibodies [donkey anti-rabbit IgG CF568 (Biotium, catalog no. 20098-1, RRID: AB_10853318) and donkey anti-chicken Alexa Fluor 647 (Jackson ImmunoResearch Labs, catalog no. 703-605-155, RRID: AB_2340379)] for 1 hour at room temperature, followed by another 3× 5-min PBS wash. Imaging was performed using a confocal laser scanning microscope (ZEISS, LSM 880) equipped with a 63×/1.4 Plan Apochromat oil immersion objective. For quantitative analysis of APC, axons and dendrites were distinguished on the basis of MAP2 signal. APC fluorescence intensity was measured only within regions exhibiting MAP2 signal above a defined threshold, and the values were subjected to statistical analysis. Statistical analyses were performed using raw data. For visualization purposes, deconvolution was applied using the deblurring function in ZEN software to generate the representative images shown in the figure.
