# Supplementary material for: The hook-like adaptor and cargo-binding (HAC) domain in the kinesin-2 tail enables adaptor assembly and cargo recognition
Source: Sci Adv. 2025 Oct 24;11(43):eady5861. doi: 10.1126/sciadv.ady5861 (PMC12551710; doi:10.1126/sciadv.ady5861)
Supplement: Supplementary file 1 — Figs. S1 to S10 Tables S1 and S2 [file sciadv.ady5861_sm.pdf]

Science Advances
AAAS

Supplementary Materials for

The hook-like adaptor and cargo-binding (HAC) domain in the kinesin-2 tail enables adaptor assembly and cargo recognition

Xuguang Jiang *et al.*

Corresponding author: Nobutaka Hirokawa, hirokawa@m.u-tokyo.ac.jp;
Masahide Kikkawa, mkikkawa@m.u-tokyo.ac.jp

Sci. Adv. **11**, eady5861 (2025)
DOI: 10.1126/sciadv.ady5861

This PDF file includes:

Figs. S1 to S10
Tables S1 and S2

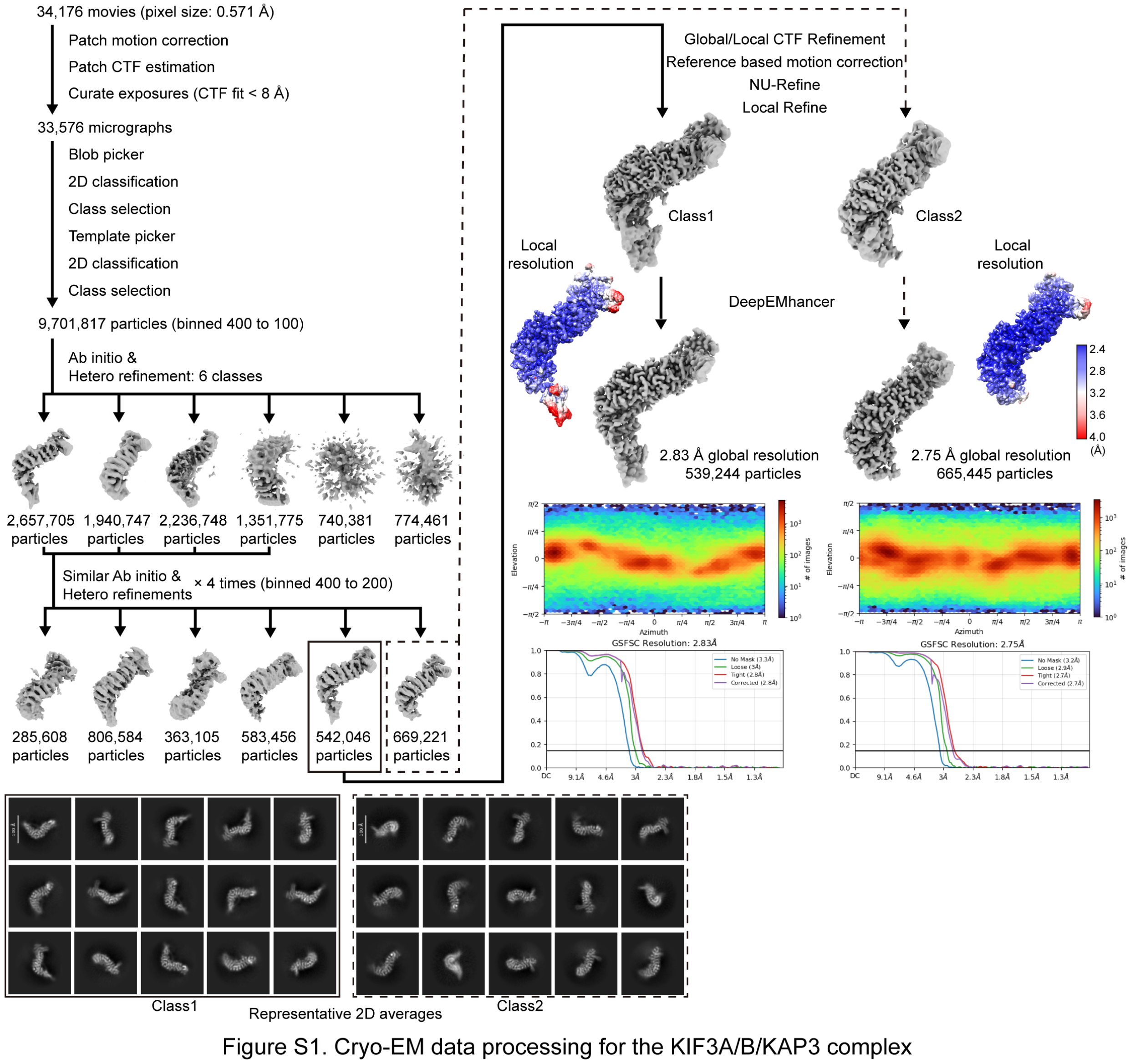

Fig. S1. Cryo-EM data processing for the KIF3A/B/KAP3 complex.

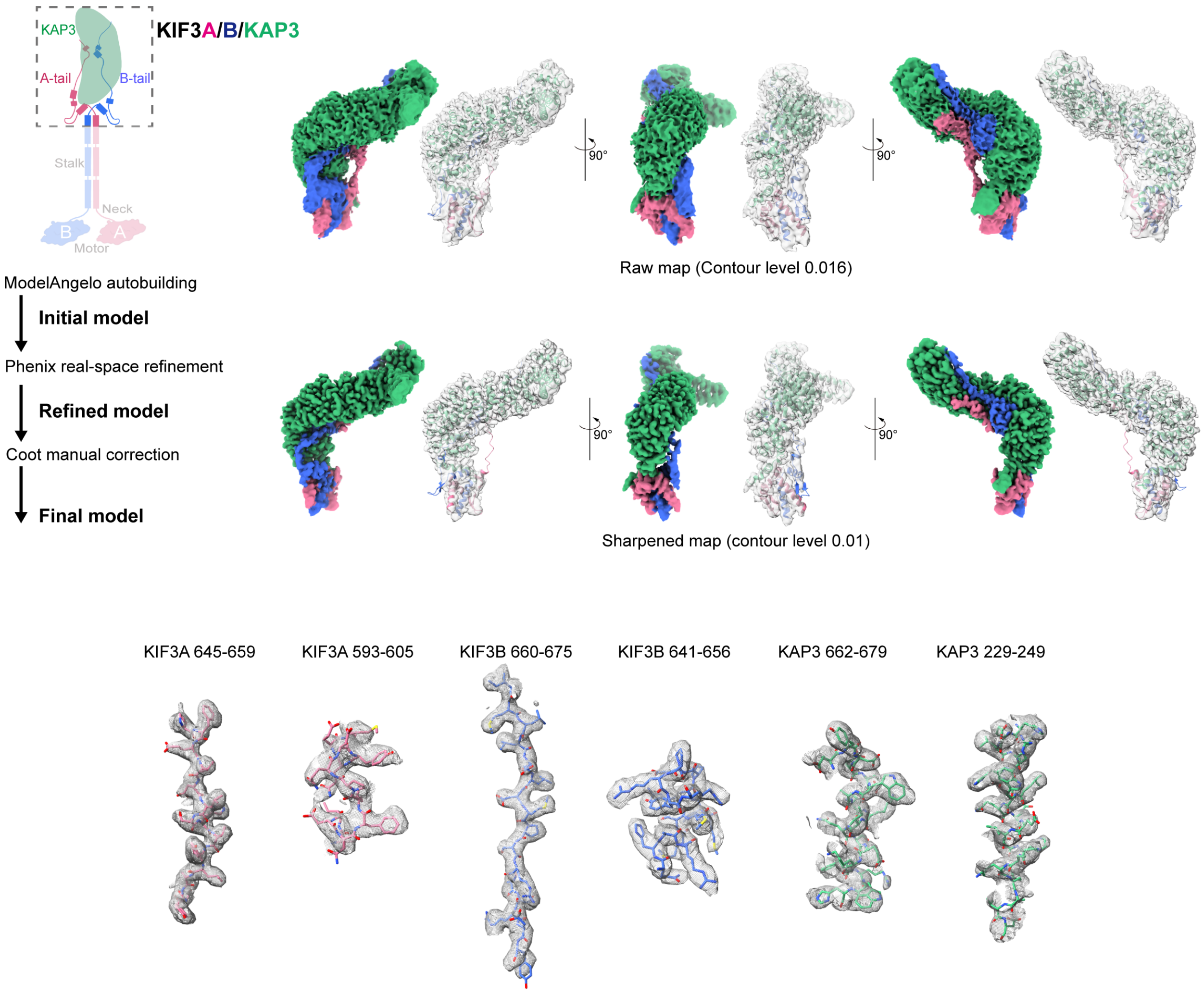

Figure S2. Model building for the KIF3A/B/KAP3 complex

Fig. S2. Model building for the KIF3A/B/KAP3 complex.

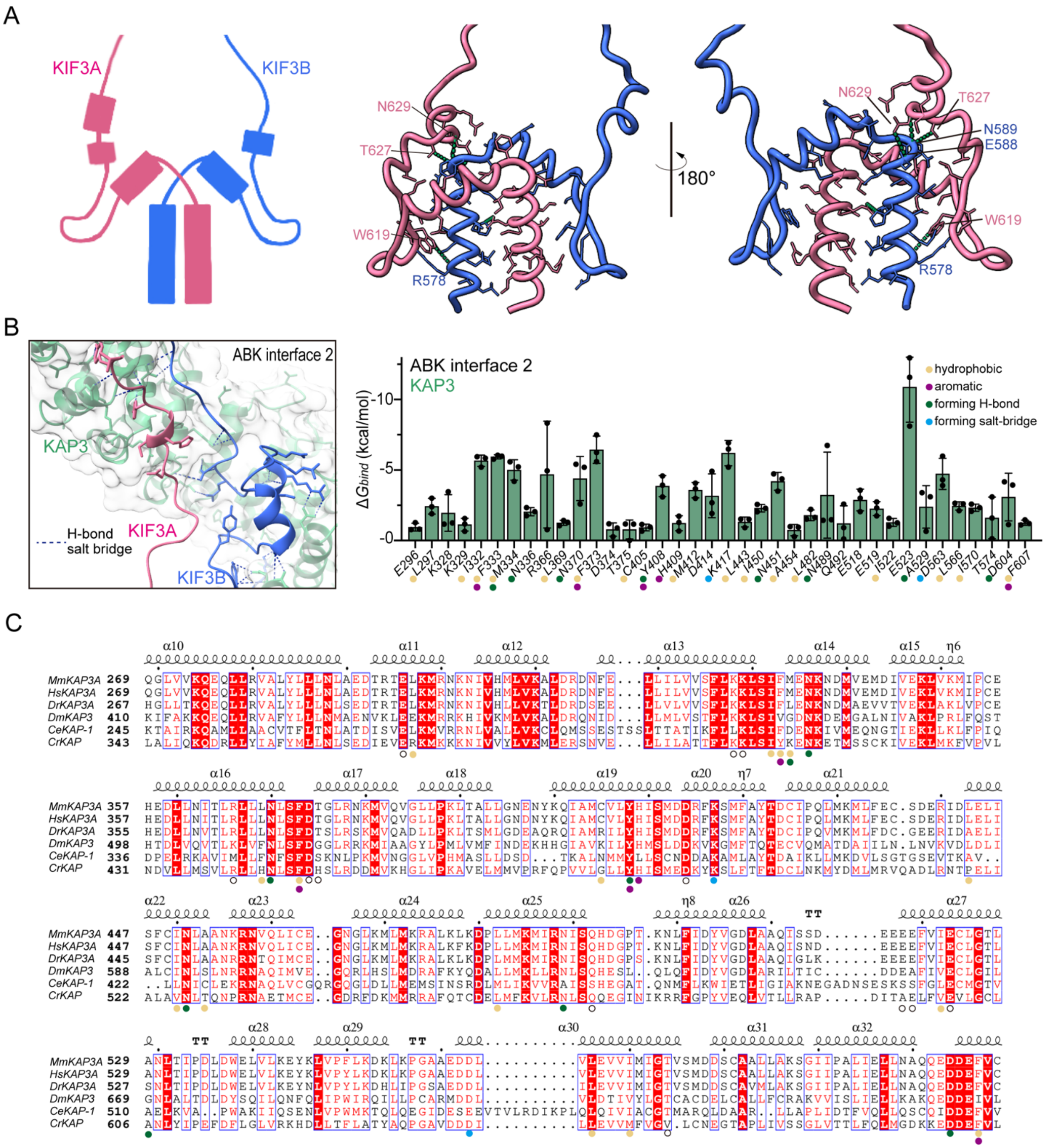

Figure S3. KIF3A/B interaction and the binding contribution of KAP3 in ABK interface 2.

Fig. S3. KIF3A/B interaction and the binding contribution of KAP3 in ABK interface 2. (**A**) Ribbon representation of the KIF3A/B stalk-tail junction. Key interacting residues and hydrogen bonds are labeled. (**B**) Cartoon representation of the ABK interface 2 structure and hydrogen bond network (left). The binding free energy contributions of KAP3 residues involved in the interface are summarized (right). Key interacting residues are labeled. (**C**) Multiple sequence alignment of KAP3 residues involved in ABK interface 2 across species. Key interacting residues are labeled.

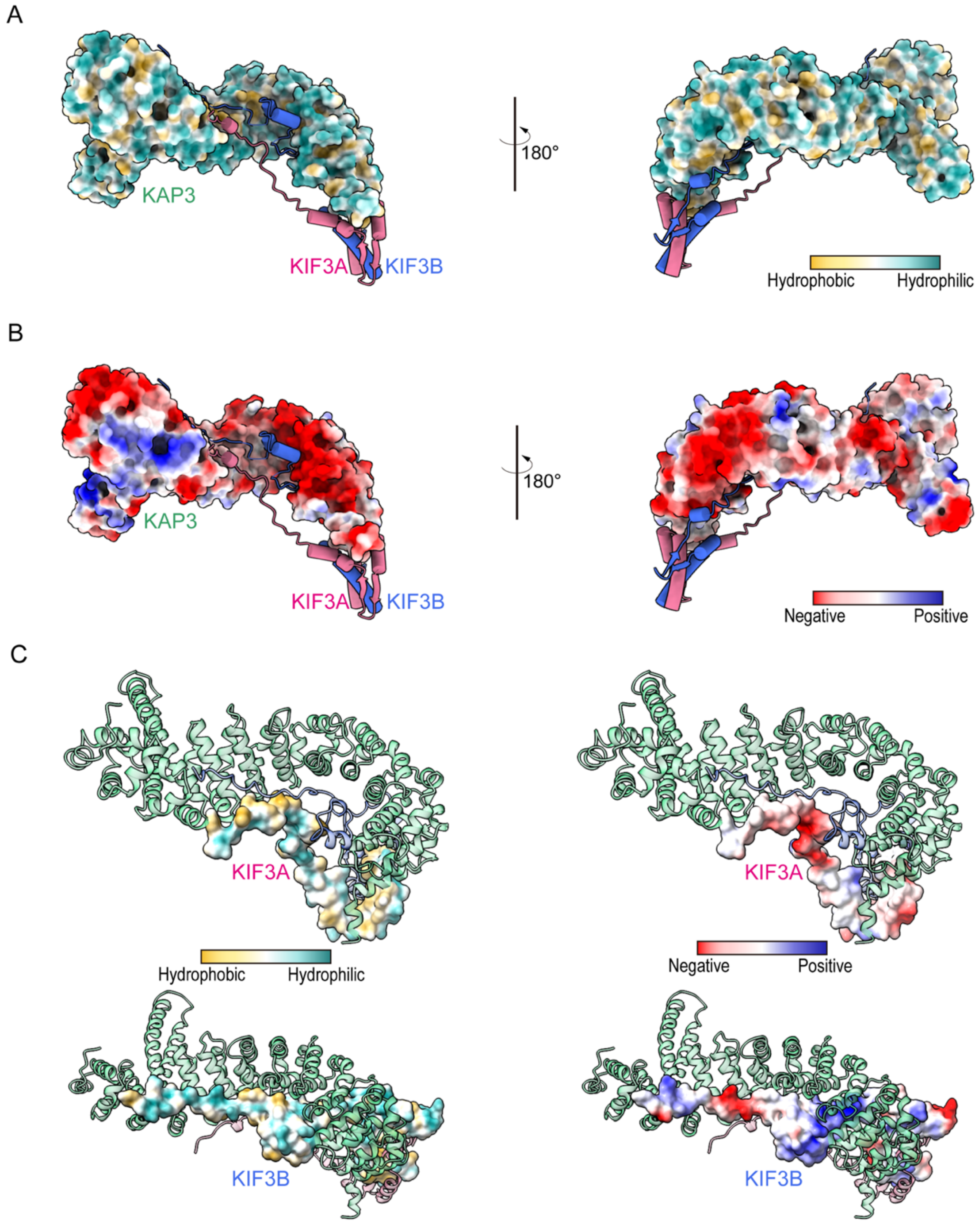

Figure S4. Surface hydrophobicity and charge of KIF3A/B and KAP3

Fig. S4. Surface hydrophobicity and charge of KIF3A/B and KAP3. (**A, B**) Hydrophobic and electrostatic properties of the KAP3 surface, with KIF3 structures shown as cartoons. (**C**) Hydrophobic and electrostatic properties of the KIF3 surface, with KAP3 structures shown as cartoons.

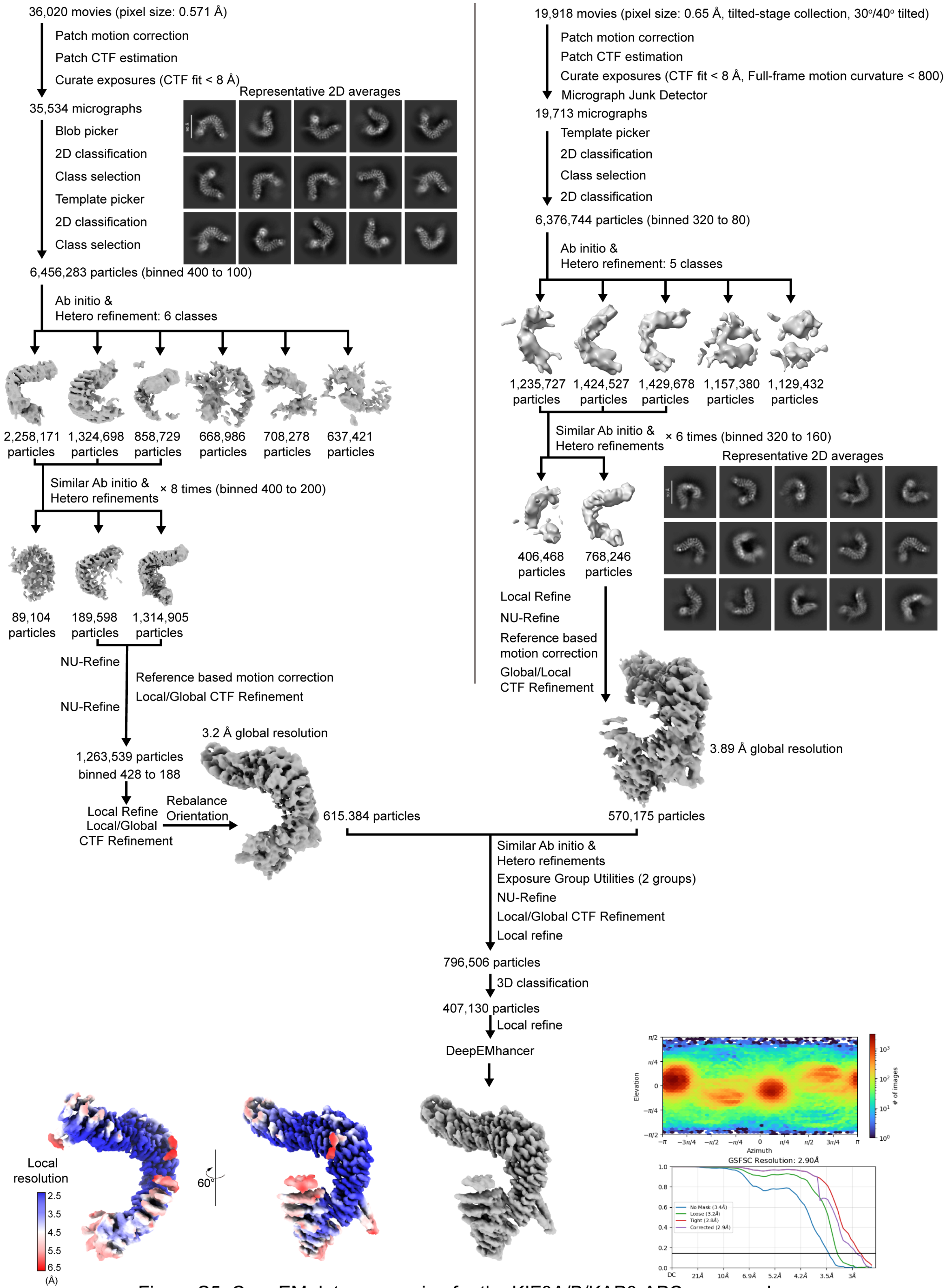

Figure S5. Cryo-EM data processing for the KIF3A/B/KAP3-APCARM complex

Fig. S5. Cryo-EM data processing for the KIF3A/B/KAP3–APC$_{ARM}$ complex.

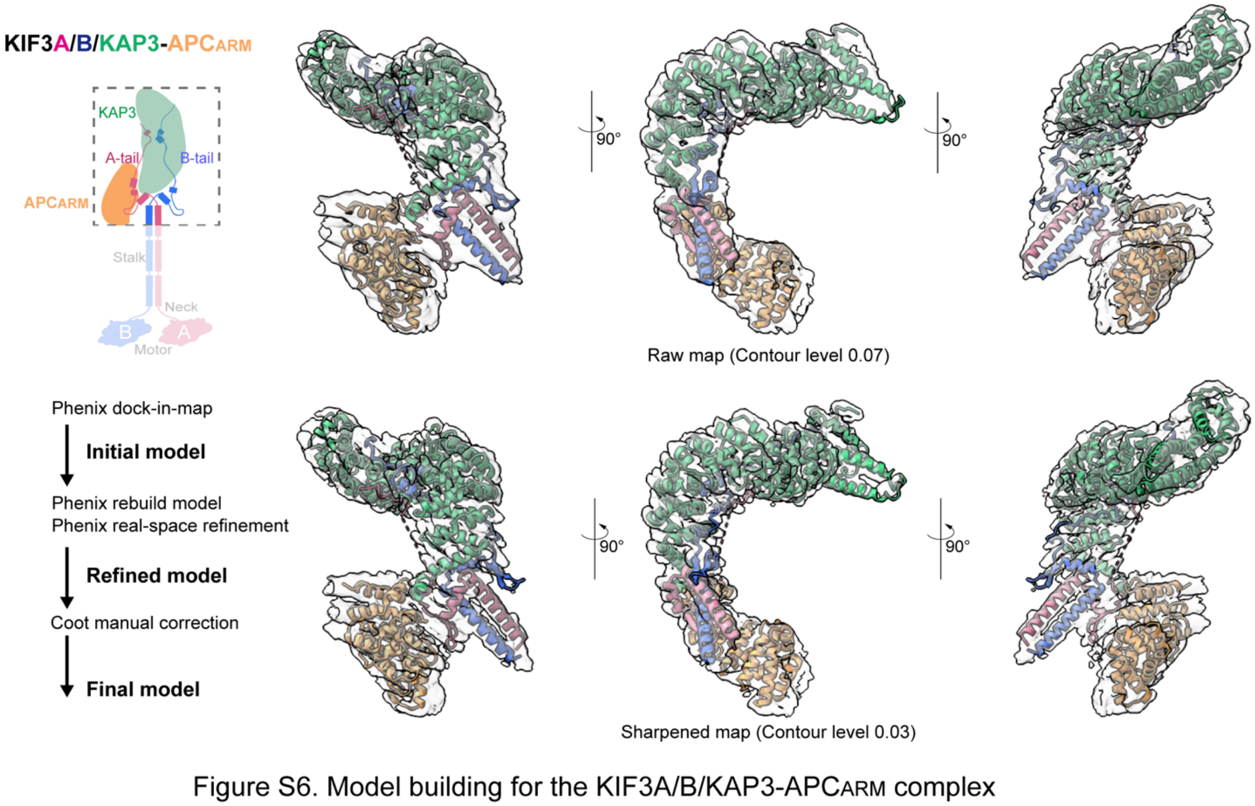

Fig. S6. Model building for the KIF3A/B/KAP3–APC$_{\text{ARM}}$ complex.

A

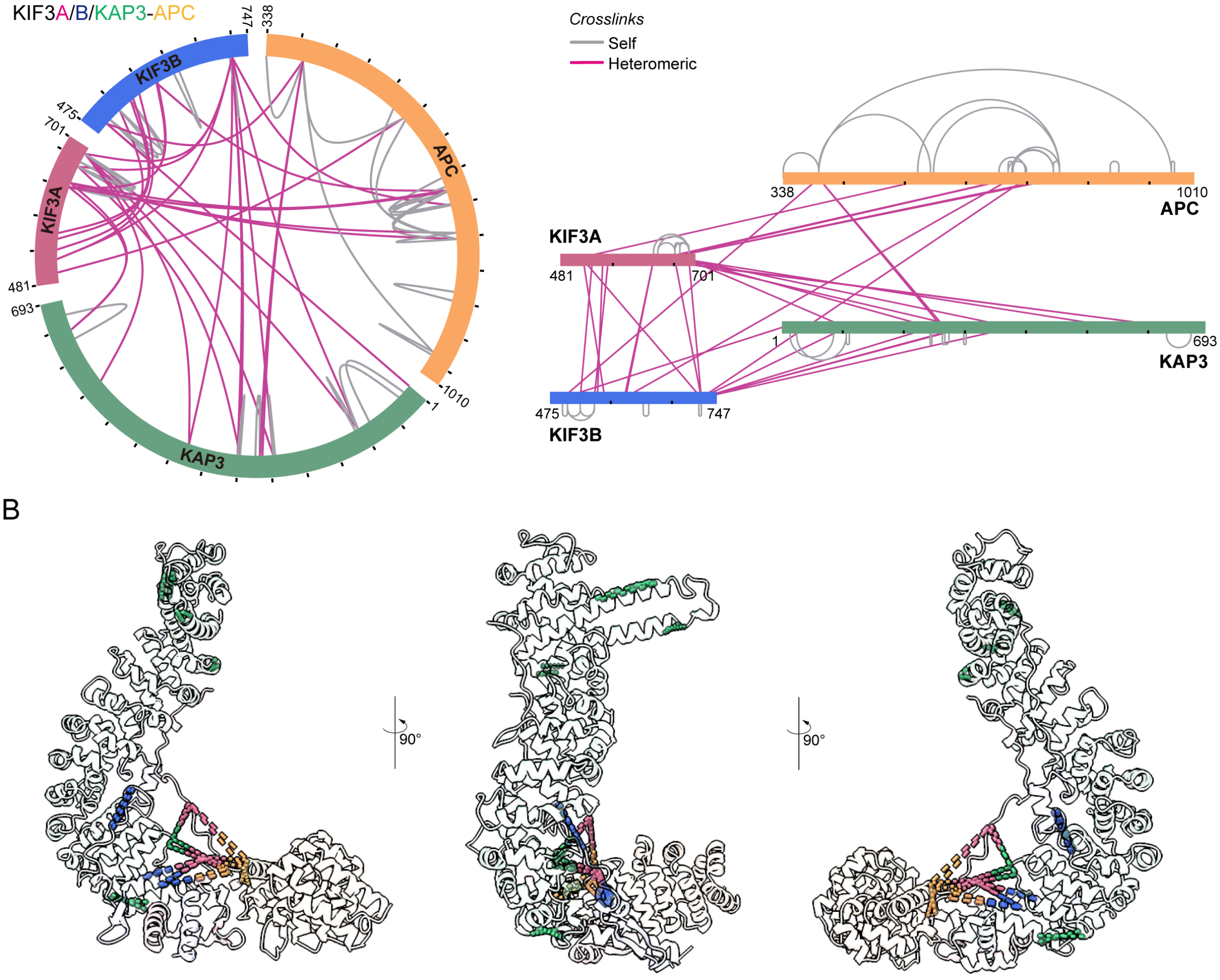

Figure S7. Cross-linking mass spectrometry (XL-MS) results of the KIF3A/B/KAP3-APC_{ARM} complex

Fig. S7. Cross-linking mass spectrometry (XL-MS) results of the KIF3A/B/KAP3–APC_{ARM} complex. (**A**) Circular representation (left) and 2D diagram (right) of XL-MS results for the ABK–APC_{ARM} complex on the constructs. Intermolecular crosslinks are shown in purple, and intramolecular crosslinks are shown in gray. Only crosslinks visible in the structural model are displayed. Full data are available in table S2. (**B**) XL-MS results mapped onto the structural model. Intermolecular crosslinks are depicted using dashed lines in two colors, each corresponding to the respective components, while intramolecular crosslinks are represented by dashed lines in a single color matching the component. Crosslink pairs are within theoretical distances.

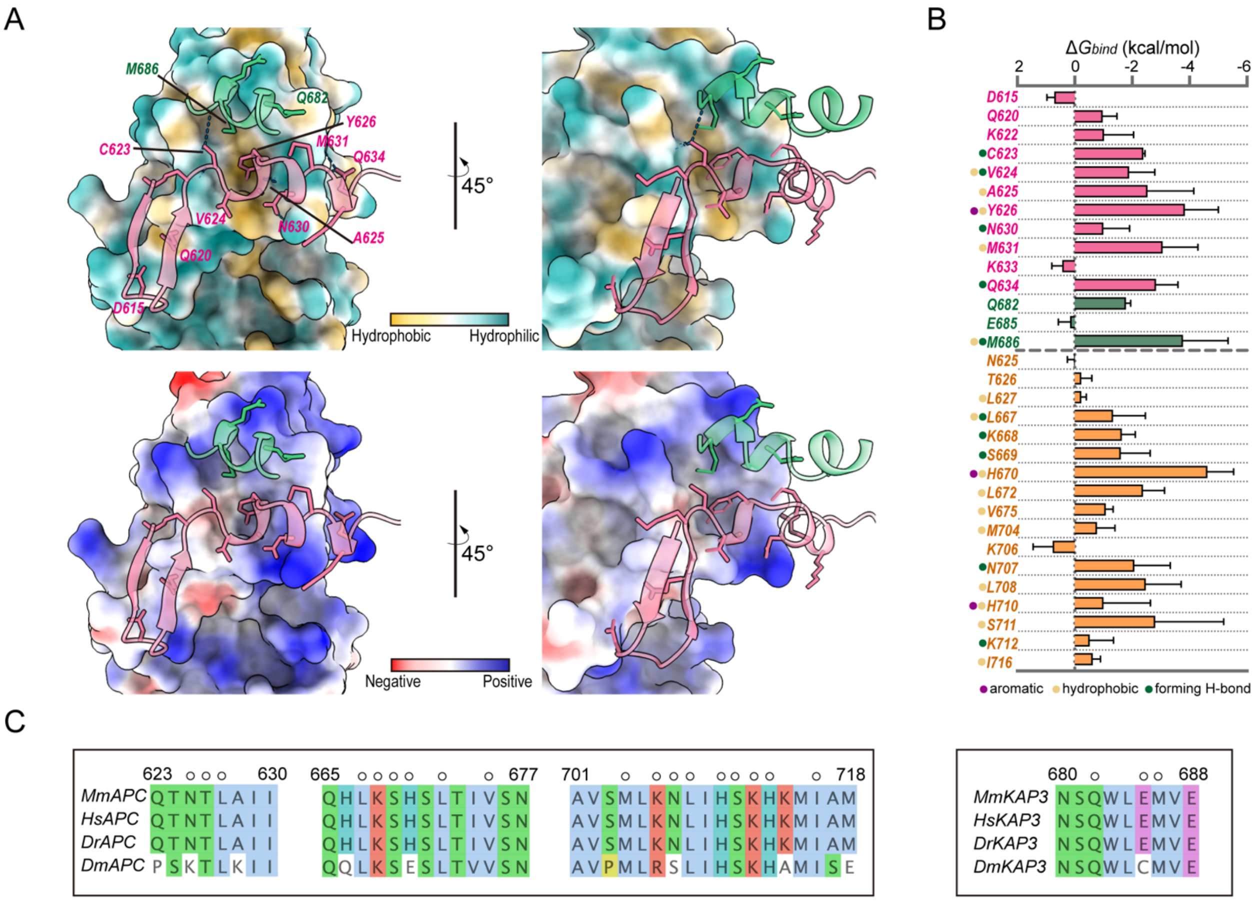

Figure S8. Molecular dynamics and MMPBSA analysis of the KIF3A/B/KAP3-APC$_{ARM}$ complex

Fig. S8. Molecular dynamics and MMPBSA analysis of the KIF3A/B/KAP3–APC$_{ARM}$ complex. (**A**) Hydrophobic (upper panel) and electrostatic (bottom panel) properties of the APC$_{ARM}$ surface, along with the cartoon structures of KIF3A/KAP3 segments involved in the interaction. The interface is primarily hydrophobic, with key interacting residues labeled. (**B**) Binding free energy contributions of residues involved in the ABKC interface. Key interacting residues and interaction patterns are labeled. Bar graphs indicate mean ± SD. (**C**) Multiple sequence alignment of KAP3 and APC residues involved in ABK-APC$_{ARM}$ binding across species. Key interacting residues are labeled. The alignment was generated using Clustal Omega (https://www.ebi.ac.uk/jdispatcher/msa/clustalo).

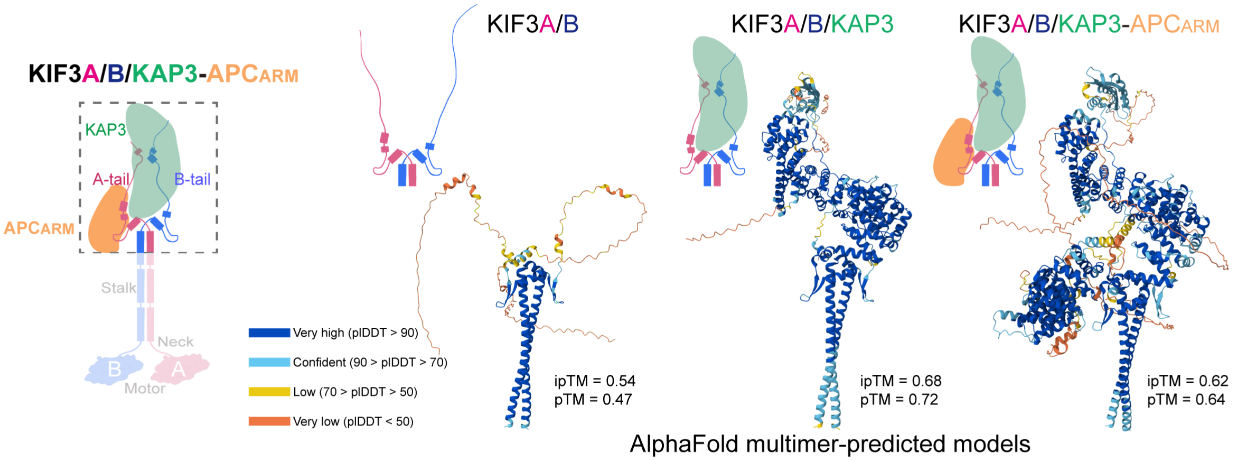

Figure S9. AlphaFold-predicted models using the full-length sequences of the constructs in this study

Fig. S9. AlphaFold-predicted models using the full-length sequences of the constructs in this study.

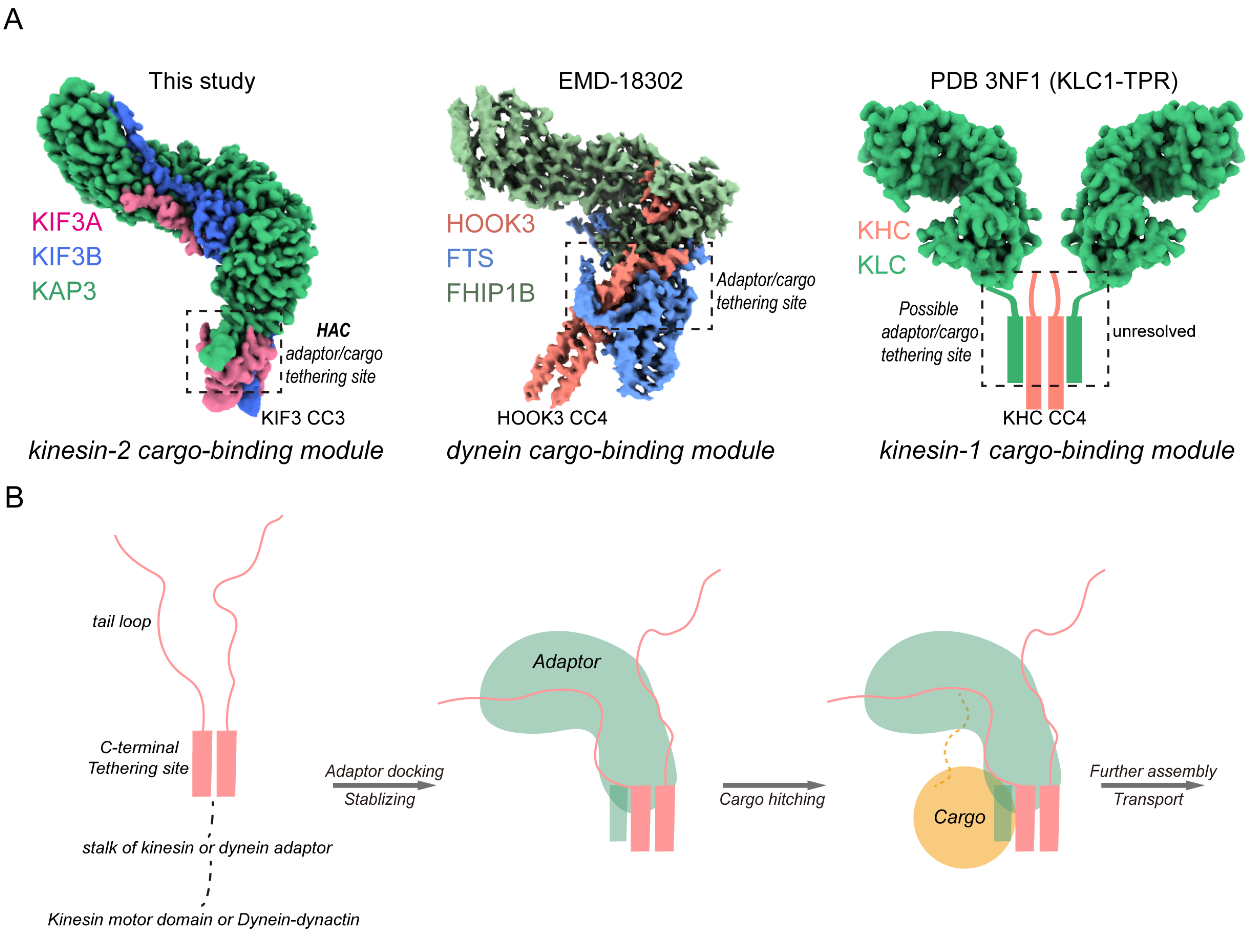

Figure S10. Hook-like cargo-binding structures suggest a shared mechanism in kinesin and dynein cargo/adaptor assembly

Fig. S10. Hook-like cargo-binding structures suggest a shared mechanism in kinesin and dynein cargo/adaptor assembly. (**A**) Comparison of the kinesin-2 ABK map obtained in this study with the dynein FTS–HOOK–FHIP1B (FTF) cargo-binding adaptor complex map and a possible kinesin-1 KHC/KLC model. The FTF density is derived from (EMDB: EMD-18302), and the density of the KLC1 TPR domain is generated based on its crystal structure (PDB: 3NF1). (**B**) A proposed common model for kinesin/dynein cargo recognition mechanism. The C-terminus of kinesin 1/2 and dynein adaptors contains an adaptor/cargo tethering site formed by helices, which mediates adaptor assembly and further recognition and binding to cargo. The form and contribution of the tethering site in cargo binding may differ; for kinesin-1 and dynein, cargo specificity is provided by the cargo-adaptor protein interaction (dashed line), while for kinesin-2, it is provided by the tethering site itself.

Table S1. Cryo-EM data collection, modeling, and refinement statistics.

	**KIF3A/B/KAP3**<br>(EMD-65777)<br>(PDB 9W9H)	**KIF3A/B/KAP3–APC$_{ARM}$**<br>(EMD-65778)<br>(PDB 9W9I)	
**Data collection and processing**			Tilted-stage collection
Microscopy	CRYO ARM 200	CRYO ARM 200	Titan Krios G3i
Detector	Gatan K3	Gatan K3	Gatan K3
Voltage (kV)	200	200	300
Cs (mm)	1.55	1.55	2.7
Tilt angle (°)	0	0	30 & 40
Slit width (eV)	20	20	20
Defocus range (μm)	0.6 ; 1.8	0.6 ; 1.8	1.0 ; 1.6
Electron exposure (*e/Å²*)	65.3	64.7	63.0
Pixel size (*Å*)	0.571	0.571	0.65
Fractions (no.)	75	80	63
Movies collected/used	34,176/33,576	36,020/35,534	19,918/19,713
Single particles (no.)	9,701,817	6,456,283	6,376,744
Single particles used (no.)	539,244	407,310	
Overall resolution (*Å*)	2.83	2.90	
FSC threshold	0.143	0.143	
**Model building and refinement**			
Model composition			
Non-hydrogen atoms	6085	7712	
Protein residues	742	1011	
R.m.s. deviation			
Bond lengths (*Å*)	0.006	0.005	
Bond angles (°)	1.105	1.087	
Validation			
MolProbity score	2.37	1.94	
Clashscore	15.68	15.50	
Poor rotamers (%)	2.49	0.88	
Ramachandran plot			
Favored (%)	94.70	96.30	
Allowed (%)	5.30	3.60	
Outliers (%)	0	0.10	

Table S2. Cross-links detected by cross-linking mass spectrometry.

Modified sequence1	Modified sequence2	Score	Crosslink type	Protein1	Protein2	Linked residue pairs
AEEQEKLLEESNMELEER	_KESTLK_	142.1	Inter-protein	KIF3A(P28741)	APC(Q61315)	K497-K579
VWTMLMAAKSEMADLQQEHQR	_LFSKLQAVK_	199.49	Inter-protein	KIF3A(P28741)	KIF3B(Q61711)	K559-K547
VWTM(Oxidation(M))LMAAKSEMADLQQEHQR	_LFSKLQAVK_	64.723	Inter-protein	KIF3A(P28741)	KIF3B(Q61711)	K559-K547
KQTPVPDK	_NKIMNR_	182.13	Inter-protein	KIF3A(P28741)	KIF3B(Q61711)	K633-K598
QSLMKLER	_TELKMR_	62.2	Inter-protein	KIF3A(P28741)	KAP3(P70188)	K668-K298
QSLMKLER	_PRTSKGK_	30.612	Intra-protein	KIF3A(P28741)	KIF3A(P28741)	K668-K676
QSLMKLERPR	_TELKMR_	64.654	Inter-protein	KIF3A(P28741)	KAP3(P70188)	K668-K298
QSLMKLERPR	_QTPVPDKK_	47.288	Intra-protein	KIF3A(P28741)	KIF3A(P28741)	K668-K640
RSAKPETVIDSLLQ	_TSKGKAR_	77.662	Intra-protein	KIF3A(P28741)	KIF3A(P28741)	K691-K676
SAKPETVIDSLLQ	_NKMVQVGLLPK_	108.71	Inter-protein	KIF3A(P28741)	KAP3(P70188)	K691-K380
SAKPETVIDSLLQ	_KAVDEDLENQTLR_	95.015	Inter-protein	KIF3A(P28741)	KAP3(P70188)	K691-K246
SAKPETVIDSLLQ	_KQTPVPDK_	89.959	Intra-protein	KIF3A(P28741)	KIF3A(P28741)	K691-K633
SAKPETVIDSLLQ	_DSLPGKEK_	70.332	Inter-protein	KIF3A(P28741)	KAP3(P70188)	K691-K106
SAKPETVIDSLLQ	_QSLMKLER_	66.004	Intra-protein	KIF3A(P28741)	KIF3A(P28741)	K691-K668
SAKPETVIDSLLQ	_LVPFLKDK_	46.746	Inter-protein	KIF3A(P28741)	KAP3(P70188)	K691-K552
SAKPETVIDSLLQ	_QTPVPDKK_	35.891	Intra-protein	KIF3A(P28741)	KIF3A(P28741)	K691-K640
MQGEDAR	_ILEQKR_	30.57	Inter-protein	KAP3(P70188)	KIF3B(Q61711)	M1-K495
_GGNIDVHPSEKALIVQYEVEATILGEMGDPMLGER	_DSLPGKEK_	75.141	Intra-protein	KAP3(P70188)	KAP3(P70188)	K25-K106
_LIHPSKLSEVEQLLYYLQNR	_VKGGNIDVHPSEK	122.53	Intra-protein	KAP3(P70188)	KAP3(P70188)	K85-K14
LIHPSKLSEVEQLLYYLQNR	_RKVKGGNIDVHPSEK_	63.573	Intra-protein	KAP3(P70188)	KAP3(P70188)	K85-K14
RDSLPGKEK	_KEKSSK_	21.993	Intra-protein	KAP3(P70188)	KAP3(P70188)	K106-K111
KAVDEDLENQTLR	_HELWQEELSKK_	229.68	Intra-protein	KAP3(P70188)	KAP3(P70188)	K246-K243
KAVDEDLENQTLR	_RHELWQEELSKK_	97.836	Intra-protein	KAP3(P70188)	KAP3(P70188)	K246-K243
KAVDEDLENQTLR	_SAKPETVIDSLLQ_	71.085	Inter-protein	KAP3(P70188)	KIF3A(P28741)	K246-K691
KYQGLVVK	_DYDKTFK_	183.24	Intra-protein	KAP3(P70188)	KAP3(P70188)	K267-K263
YQGLVVKQEQLLR	_DYDKTFK_	60.628	Intra-protein	KAP3(P70188)	KAP3(P70188)	K274-K263
NKNIVHMLVK	_TELKMR_	220.51	Intra-protein	KAP3(P70188)	KAP3(P70188)	K302-K298
NKNIVHMLVK	_TELKM(Oxidation(M))R_	169.12	Intra-protein	KAP3(P70188)	KAP3(P70188)	K302-K298
NKNIVHM(Oxidation (M))LVK	_TELKMR_	133.57	Intra-protein	KAP3(P70188)	KAP3(P70188)	K302-K298
DVIIKETQAPAYLIDLMHDK	_KIQSEK_	191.49	Intra-protein	KAP3(P70188)	KAP3(P70188)	K630-K670
ETQAPAYLIDLMHDKNNEIR	_KQTPVPDK_	77.505	Inter-protein	KAP3(P70188)	KIF3A(P28741)	K645-K633
ETQAPAYLIDLMHDKNNEIR	_QTPVPDKK_	56.238	Inter-protein	KAP3(P70188)	KIF3A(P28741)	K645-K640
ASAALHNIIHSQPDDKR	_DYDKTFK_	141.46	Inter-protein	APC(Q61315)	KAP3(P70188)	K396-K263
ASAALHNIIHSQPDDKR	_KESTLK_	141.32	Intra-protein	APC(Q61315)	APC(Q61315)	K396-K579
ASAALHNIIHSQPDDKR	_ILEQKR_	116.51	Inter-protein	APC(Q61315)	KIF3B(Q61711)	K396-K495
ASAALHNIIHSQPDDKR	_KYQGLVVK_	79.346	Inter-protein	APC(Q61315)	KAP3(P70188)	K396-K338
ASAALHNIIHSQPDDKR	_ASAALHNIIHSQPDDKR_	37.013	Intra-protein	APC(Q61315)	APC(Q61315)	K396-K267

ESTLKSVLSALWN LSAHCTENK	_HKQNLYGDYAF DANR_	17.619	Intra-protein	APC(Q61315)	APC(Q61315)	K584-K790
NPKDQEALWDMG AVSMLK	_NLMANRPAKYK _	129.05	Intra-protein	APC(Q61315)	APC(Q61315)	K691-K734
DQEALWDMGAVS MLKNLIHSK	_YKDANIMSPGSS LPSLHVR_	71.085	Intra-protein	APC(Q61315)	APC(Q61315)	K706-K736
DQEALWDMGAVS MLKNLIHSK	_HKMIAMGSAAA LR_	65.207	Intra-protein	APC(Q61315)	APC(Q61315)	K706-K714
NLIHSKHK	_KQTPVPDK_	122.52	Inter-protein	APC(Q61315)	KIF3A(P28741)	K712-K633
HKMIAMGSAAAL R	_KQTPVPDK_	134.49	Inter-protein	APC(Q61315)	KIF3A(P28741)	K714-K633
HKMIAMGSAAAL R	_QTPVPDKK_	101.6	Inter-protein	APC(Q61315)	KIF3A(P28741)	K714-K640
ALEAELDAQHLSE TFDNIDNLSPKASH R	_HKMIAMGSAAA LR_	209.38	Intra-protein	APC(Q61315)	APC(Q61315)	K780-K714
ALEAELDAQHLSE TFDNIDNLSPKASH R	_HKQNLYGDYAF DANR_	158.94	Intra-protein	APC(Q61315)	APC(Q61315)	K780-K790
ALEAELDAQHLSE TFDNIDNLSPKASH R	_QTPVPDKK_	95.138	Inter-protein	APC(Q61315)	KIF3A(P28741)	K780-K640
ALEAELDAQHLSE TFDNIDNLSPKASH R	_HKM(Oxidation (M))IAMGSAAAL R	77.51	Intra-protein	APC(Q61315)	APC(Q61315)	K780-K714
ALEAELDAQHLSE TFDNIDNLSPKASH R	_HKMIAM(Oxidati on (M))GSAAALR_	50.492	Intra-protein	APC(Q61315)	APC(Q61315)	K780-K714
ALEAELDAQHLSE TFDNIDNLSPKASH R	_NLIHSKHK_	37.44	Intra-protein	APC(Q61315)	APC(Q61315)	K780-K712
HKQNLYGDYAFD ANR	_HKMIAMGSAAA LR_	160.47	Intra-protein	APC(Q61315)	APC(Q61315)	K790-K714
HKQNLYGDYAFD ANR	_QTPVPDKK_	90.334	Inter-protein	APC(Q61315)	KIF3A(P28741)	K790-K640
HKQNLYGDYAFD ANR	_ADVNSKK_	67.115	Intra-protein	APC(Q61315)	APC(Q61315)	K790-K558
HKQNLYGDYAFD ANR	_HKM(Oxidation(M))IAMGSAAALR_	48.288	Intra-protein	APC(Q61315)	APC(Q61315)	K790-K714
GLQITTTAAQIAK VMEEVSAIHTSQDD R	_GIGLSAYHPTTE NAGTSSKR_	81.179	Intra-protein	APC(Q61315)	APC(Q61315)	K887-K873
SSNDSLNSVTSSD GYGKR	_GQMKPSVESYS EDDESK_	85.884	Intra-protein	APC(Q61315)	APC(Q61315)	K973-K978
SSNDSLNSVTSSD GYGKR	_ASAALHNIIHSQ PDDKR_	34.474	Intra-protein	APC(Q61315)	APC(Q61315)	K973-K396
NIVDHTNEQQKIL EQKR	_QEIAEQKRR_	64.565	Intra-protein	KIF3B(Q61711)	KIF3B(Q61711)	K495-K503
QEIAEQKR	ILEQKR	175.75	Intra-protein	KIF3B(Q61711)	KIF3B(Q61711)	K503-K495
DEETLELKETYTS LQQEVDIK	_LDIEEKYTSLQE EAQGK_	145.13	Inter-protein	KIF3B(Q61711)	KIF3A(P28741)	K524-K533
_DEETLELKETYTS LQQEVDIK	_QEIAEQKR_	103.66	Intra-protein	KIF3B(Q61711)	KIF3B(Q61711)	K524-K503
DEETLELKETYTS LQQEVDIK	_LFSKLQAVK_	98.165	Intra-protein	KIF3B(Q61711)	KIF3B(Q61711)	K524-K547
LFSKLQAVK	_QEIAEQKR_	55.353	Intra-protein	KIF3B(Q61711)	KIF3B(Q61711)	K547-K503
LQAVKAEIHDLQE EHIK	_KVWTMLMAAK _	188.31	Inter-protein	KIF3B(Q61711)	KIF3A(P28741)	K552-K550
_AEIHDLQEEHIKER _	_KELEEK_	125.97	Inter-protein	KIF3B(Q61711)	KIF3A(P28741)	K564-K518
_HLIIENFIPLEEKNK _	_KQTPVPDK_	110.92	Inter-protein	KIF3B(Q61711)	KIF3A(P28741)	K596-K633
_HLIIENFIPLEEKNK _	_NLIHSKHK_	96.591	Inter-protein	KIF3B(Q61711)	APC(Q61315)	K596-K712
PVSAVGYKR	LENQQMMKR	37.18	Intra-protein	KIF3B(Q61711)	KIF3B(Q61711)	K636-K627
KSGSSSSSSGNPAS QFYPQSR	_KAVDEDLENQT LR_	145.75	Inter-protein	KIF3B(Q61711)	KAP3(P70188)	K722-K246

KSGSSSSSSGNPAS QFYPQSR	_KELEEK_	122.75	Inter-protein	KIF3B(Q61711)	KIF3A(P28741)	K722-K518
KSGSSSSSSGNPAS QFYPQSR	_NKMVQVGLLPK _	107.45	Inter-protein	KIF3B(Q61711)	KAP3(P70188)	K722-K380
KSGSSSSSSGNPAS QFYPQSR	_TELKMR_	106.28	Inter-protein	KIF3B(Q61711)	KAP3(P70188)	K722-K298
KSGSSSSSSGNPAS QFYPQSR	_ARPKSGR_	94.203	Intra-protein	KIF3B(Q61711)	KIF3B(Q61711)	K722-K718
KSGSSSSSSGNPAS QFYPQSR	_QSLMKLERPR_	75.533	Inter-protein	KIF3B(Q61711)	KIF3A(P28741)	K722-K668
KSGSSSSSSGNPAS QFYPQSR	_DSLPGKEK_	72.421	Inter-protein	KIF3B(Q61711)	KAP3(P70188)	K722-K106
KSGSSSSSSGNPAS QFYPQSR	_NKNIVHMLVK_	67.251	Inter-protein	KIF3B(Q61711)	KAP3(P70188)	K722-K302
KSGSSSSSSGNPAS QFYPQSR	_SAKPETVIDSLL Q_	58.373	Inter-protein	KIF3B(Q61711)	KIF3A(P28741)	K722-K691
KSGSSSSSSGNPAS QFYPQSR	_YKDANIMSPGSS LPSLHVR_	49.101	Inter-protein	KIF3B(Q61711)	APC(Q61315)	K722-K736
